# Comparative Analysis of Microbial Community Diversity and Dynamics on Diseased Tubers During Potato Storage in Different Regions of Qinghai China

**DOI:** 10.3389/fgene.2022.818940

**Published:** 2022-02-22

**Authors:** Tianyan Xie, Shuo Shen, Yufan Hao, Wei Li, Jian Wang

**Affiliations:** ^1^ Academy of Agriculture and Forestry Sciences, Qinghai University, Xining, China; ^2^ Key Laboratory of Potato Breeding of Qinghai Province, Xining, China; ^3^ State Key Laboratory of Plateau Ecology and Agriculture, Qinghai University, Xining, China; ^4^ Key Laboratory of Qinghai Tibet Plateau Biotechnology, Ministry of Education, Xining, China; ^5^ Northwest Potato Engineering Research Center, Ministry of Education, Xining, China

**Keywords:** pre-storage treatment, microbial community, potato storage, phytopathogenic microbe, high-throughput sequencing

## Abstract

Effective storage of potatoes is very important for the food industry. Given the problems involving rotten potatoes and low quality during storage, harvested potatoes from the main potato-producing areas in the Qinghai Plateau were treated by selection and air drying (Group “A”) and the others were stored directly as controls (Group “C”). Then, the microbial community structure and diversity of diseased potato tubers from four main production areas were analyzed by high-throughput sequencing technology in different storage stages. The results showed that the community composition and diversity of microbes in different regions and storage periods were different, and the dominant fungi in diseased potato tubers were *Boeremia* in Huangyuan (HY), Maying (MY) and Zhongling (ZL) and *Apiotrichum* in Huangzhong (HZ) at the genus level. The dominant bacterial genus was *Pseudomonas*, but its abundance varied in samples from different regions and storage periods. In the analysis of indicator species, there were some common species and endemic species in each region and period, and the period with the largest number of different species was the third period. Among the four storage periods, the region with the largest number of different species was HZ. Some fungi, especially *Fusarium* and other potato pathogens, were more abundant in control Group “C” than in treatment Group “A.” In the diversity analysis, the *α* diversity of fungi in Group “C” was higher than that in Group “A,” but the *α* diversity of bacteria in Group “A” was higher than that in Group “C,” and there was no obvious regularity with storage time. The *β* diversity varied significantly among different regions. In addition, through functional prediction analysis, it was found that a plant pathogen was one of the main nutritional types of fungi, which indicated that treatment by selection and drying could significantly reduce phytopathogenic microbe and other microorganisms and could be used as an effective measure for potato storage compared with the prevention and control by drugs that can cause environmental pollution. Further analysis of co-occurrence network showed that pathogenic fungi *Fusarium* was negatively correlated with pathogenic bacteria *Erwinia*, and there is also a negative correlation between pathogens and antagonistic microorganisms indicated that there were various symbiotic relationships among microorganisms in diseased potatoes. This study may provide a theoretical basis for biological control of potato cellar diseases and the maintenance of potato quality during long-term storage.

## 1 Introduction

Potato (*Solanum tuberosum*) is a staple of the world’s diet and is grown in more than 100 countries. It is the fourth largest crop after corn, wheat and rice (Boivin et al., 2020). At present, the yield of potato is much higher than that of wheat, corn and rice, so there is room for improvement. Compared with other staple grain crops, the development of the potato industry will become increasingly important in ensuring world food security in the future. However, there are also many diseases during the growing and storage period of potatoes, such as the brown rot caused by *Ralstonia solanacearum* (Norman et al., 2020), dry rot caused by *Fusarium* (Stefańczyk and Sobkowiak, 2017), Black dot caused by *Colletotrichum coccodes* (Lees and Hilton, 2003), Black Scurf on potato tubers and Stem Canker caused by *Rhizoctonia solani* (Yang and Wu, 2012; Takooree et al., 2021), leaf wilt and postharvest tuber rot caused by *Galactomyces candidum* (Song et al., 2020), early blight and late blight caused by *Alternaria* and *Phytophthora* (Wharton et al., 2012; Gevens and Seidl, 2013; Guo et al., 2021; Takooree et al., 2021), wart disease caused by *Synchytrium endobioticum* (Bonants et al., 2015) and so on. The planting area and total output of potato in China ranks first in the world, but there are still many limiting factors in production, such as the poor quality of seed potatoes, simple storage methods, and numerous rotten potatoes (Li Yang et al., 2020).

The effective storage of potato tubers is very important to maintain the quality attributes of fresh tubers, and it is a key step for the potato industry to carry out regular research (Vreugdenhil et al., 2007). A good potato storage facility must be able to control temperature and humidity and be equipped with a good ventilation system. As potatoes may be damaged during harvest, potatoes are usually prestored at a high relative humidity of 10–15°C (95%) for approximately 2 weeks. The pre-storage conditions can make the potato dry and heal skin damage. After pre-storage, the potato can be stored at a lower temperature for several weeks to months (Pinhero et al., 2009), so the temperature plays a role in resistance, with high temperatures usually increasing the severity of disease and cool temperatures promoting the occurrence of asymptomatic plants (Norman et al., 2020). In addition, some studies have shown that UV-C can improve the resistance of potato tubers to *Fusarium* rot and detect germination activity in the early stage to realize advanced auxiliary potato storage management (Stevens et al., 1999; Jakubowski and Królczyk, 2020; Rady et al., 2020).

High-throughput sequencing can produce sequencing data that are accurate and reflect the actual situation of microbial diversity, so the approach is widely used in the study of microbial diversity (Williams et al., 2014; Schirmer et al., 2015) and changes in microbial communities such as consumption of milk, fish fillets, yellow-feathered broilers and other foods during storage (Porcellato et al., 2018; Teresa et al., 2019; Wang et al., 2020). At present, the study of potato microbial diversity by high-throughput sequencing is mainly focused on rhizosphere soil in the field, including the study of fungal diversity in rhizosphere soil of continuous cropping potato subjected to different furrow-ridge mulching management approaches (Qin et al., 2017), temporal shifts of fungal communities in the rhizosphere and on tubers in potato fields (Zimudzi et al., 2018), and the rhizosphere microbial diversity and community dynamics during potato cultivation (Hou et al., 2020). With regard to plant pathogens, comparative analysis showed that the abundance of *Fusarium* was significantly different with different varieties of crop rotation, and it was possible to control plant pathogenic fungi by rotation management (Mardanova et al., 2019). In potato storage, only the microbial diversity of fresh-cut potatoes in different storage periods was studied (Kong et al., 2020), so there is less research on microbial diversity related to potato diseases. Only the community structure and diversity of fungi and bacteria in potato scab spots was studied, which showed that *Streptomyces*, the pathogen of potato scab, was present in all scab samples (Wang, 2017).

In this study, the Illumina MiSeq high-throughput sequencing technique was used to study the microbial community structure and diversity of potato disease spots infected by pathogens during storage. We analyzed the differences in microbial community composition and diversity of potato disease spots treated by selection and air drying compared with the control in different storage periods and different ecological regions to identify the suspected pathogenic microorganisms causing fungal and bacterial diseases of potato. The interaction between pathogenic microorganisms and antagonistic bacteria was studied, and the microbial structure and community diversity of potato diseases in different regions and treatments were discussed. The purpose of this paper is to clarify the microbial community structure of diseased potato tubers, identify the pathogenic microbes of potato and the interaction between antagonistic microorganism and pathogens. At the same time, the positive effects of the selection and air-drying treatment before entering the cellar on potato cellar diseases were determined, which provideed a theoretical basis for the effective storage and production of potatos. It is expected to provide theoretical reference for the biological control of potato cellar diseases in Qinghai, ensure the quality of potato during storage, reduce drug residues and environmental pollution caused by chemical control, protect the ecological environment, and provide technical support for effective potato storage, which is of great significance for the development of potato industry.

## 2 Materials and Methods

### 2.1 Sampling

The potato variety in our experiment is Q 9, which is widely planted in Northwest China, and the samples included in this study were collected from the main producing areas of potato: HY, MY, ZL, and HZ (Qinghai Province, China). For the potatoes in HY, MY and ZL, after harvest, healthy potatoes after selecting diseased, rotten and damaged tubers were prestored in a dry and ventilated place under natural conditions to air-dry avoid direct sunlight for 7 days before entering the cellar as a treatment (Group “A”), and the rest of the potatoes, including potatoes in HZ, went directly into the cellar as a control (Group “C”). Potato samples with diseased spots are judged according to the symptoms and the types of disease, and collected on Days 0, 40, 80 and 120 during the storage period of 120 days (periods 1, 2, 3, and 4). Two to 5 g of the diseased potato tubers was obtained from each group of samples, with five repeats. A total of 25 sample groups were immediately stored at −80°C for subsequent analysis (Table 1).

**TABLE 1:** Information on samples of diseased potato tubers during storage in different regions.

Region	Geographic coordinates	Altitude (m)	Size of the cellar (m^2^)	Treatment	Temperature, humidity and samples of each period											
					Period 1			Period 2			Period 3			Period 4		
					T (°C)	H (%)	Samples	T (°C)	H (%)	Samples	T (°C)	H (%)	Samples	T (°C)	H (%)	Samples
HY	101°04′12″E	3,119	150	Air Drying control	6.00	85.00	none	5.10	99.00	HY-2-A	2.90	99.00	HY-3-A	4.30	99.00	HY-4-A
	36°73′31″N						HY-1-C			HY-2-C			HY-3-C			HY-4-C
MY	102°66′23″E	2,773	60	Air Drying control	5.10	86.00	none	2.00	90.00	MY-2-A	0.30	98.00	MY-3-A	3.80	99.00	MY-4-A
	36°55′82″N						MY-1-C			MY-2-C			MY-3-C			MY-4-C
ZL	102°51′21″E	2,685	90	Air Drying control	5.70	80.00	none	4.20	99.00	ZL-2-A	2.40	99.00	ZL-3-A	4.00	99.00	ZL-4-A
	36°54′70″N						ZL-1-C			ZL-2-C			ZL-3-C			ZL-4-C
HZ	101°37′15″E	2,610	300	control	5.50	88.00	HZ-1-C	2.10	99.00	HZ-2-C	−1.40	99.00	HZ-3-C	4.90	99.00	HZ-4-C
	36°40′44″N															

### 2.2 DNA Extraction and PCR Amplification

Microbial DNA was extracted using HiPure Soil DNA Kits (Magen, Guangzhou, China) according to the manufacturer’s protocols. The V5–V7 region of the 16S rRNA was amplified using primers 799F (5′-AACMGGATTAGATACCCKG-3′) and 1193R (5′-ACG​TCA​TCC​CCA​CCT​TCC-3′) (Beckers et al., 2016) by PCR under conditions of 95°C for 5 min, followed by 30 cycles at 95°C for 1 min, 60°C for 1 min, and 72°C for 1 min and a final extension at 72°C for 7 min and the internal transcribed spacer 1 (ITS 1) region of fungi amplicons was amplified with ITS1_F_KYO2 (5′-TAG​AGG​AAG​TAA​AAG​TCG​TAA-3′) and ITS86R (5′-TTC​AAA​GAT​TCG​ATG​ATT​CAC-3′) primers (Scibetta et al., 2018) by PCR under conditions of 94°C for 2 min, followed by 30 cycles at 98°C for 10 s, 62°C for 30 s, and 68°C for 30 s and a final extension at 68°C for 5 min. PCRs were performed in triplicate with a 50 μL mixture containing 10 μL of 5 × Q5@ Reaction Buffer, 10 μL of High Fidelity@ High GC Enhancer, 1.5 μL of 2.5 mM dNTPs, 1.5 μL of each primer (10 μM), 0.2 μL of Q5@ High GC Enhancer, and 1.5 μL of template DNA template. Related PCR reagents were from New England Biolabs, United States. Purified amplicons were pooled in equimolar amounts and paired-end sequenced (PE250) on an Illumina platform according to standard protocols.

### 2.3 Bioinformatics Analysis

Raw reads were filtered using FASTP (Chen et al., 2018) (version 0.18.0), and paired-end clean reads were merged as raw tags using FLSAH (Mago et al., 2011) (version 1.2.11). Noisy sequences of raw tags were filtered under specific filtering conditions (Bokulich et al., 2013) to obtain high-quality clean tags and then clustered into operational taxonomic units (OTUs) of ≥97% similarity using the UPARSE (Edgar, 2013) (version 9.2.64) pipeline. All chimeric tags were removed using the UCHIME algorithm (Edgar et al., 2011). The representative OTU sequences were classified into organisms by a naive Bayesian model using the RDP classifier (Wang et al., 2007) (version 2.2) based on the SILVA database (Pruesse et al., 2007) (version 132), UNITE database (Nilsson et al., 2019) (version 8.0) or ITS2 database (Ankenbrand et al., 2015) (version update_2015), with a confidence threshold value of 0.8. The relative abundance of the species were calculated (Relative abundance of a species = Total effective tags of this species in a sample/All tags of this sample × 100%) and the stacked bar plot of the community composition was visualized in the R project ggplot2 package (version 2.2.1) (Wickham and Chang, 2015), and the circular layout representations of species abundance were graphed using circos (version 0.69–3) (Krzywinski et al., 2009). Venn analysis of the microbial community between groups in different regions was performed in the R project Venn Diagram package (Chen and Boutros, 2011) (version 1.6.16). The biomarker features in each group were screened by Linear discriminant analysis Effect Size (LEfSe) software (Segata et al., 2011) (version 1.0). A heatmap of species abundance was plotted using the pheatmap package (version 1.0.12) (Kolde, 2015). Chao1, ACE, Shannon, Simpson, Good’s coverage and Pielou’s evenness index were calculated in QIIME (Caporaso et al., 2010), and comparisons between groups were calculated by Welch’s *t*-test. Comparisons among groups were computed by the Kruskal–Wallis H test in the R project Vegan package (version 2.5.3) (Oksanen et al., 2010). The differences in the fungal and bacterial community compositions were compared using nonmetric multidimensional scaling (NMDS) based on the Bray–Curtis distance (version 2.5.3), and the Analysis of similarities (ANOSIM) test was calculated in the R project Vegan package. The functional group (guild) of the fungi was inferred using FUNGuild (Nguyen et al., 2016) (version 1.0), the Kyoto Encyclopedia of Genes and Genomes (KEGG) pathway analysis of bacteria was inferred using Phylogenetic Investigation of Communities by Reconstruction of Unobserved States (PICRUSt) (Langille et al., 2013) (version 2.1.4), and the ecological functional profiles were generated using the Functional Annotation of Prokaryotic Taxa (FAPROTAX) database and associated software (version 1.0). Pearson correlation analysis of species was calculated in R project psych package (version 1.8.4). Network of correlation coefficient were generated using Omicsmart and igraph package.

### 2.4 Nucleotide Sequence Accession Numbers

Sequences reported in this paper are available in the NCBI Sequence Read Archive (SRA) database under accession numbers SRR16771893–SRR16772017, (bacterial 16S rRNA gene sequences) and SRR16643766–SRR16643890 (fungi ITS 1 region sequences).

## 3 Results

### 3.1 Analysis of the Illumina Sequencing Data

We generated a total of 16,207,492 ITS raw sequences (averaging 124,673 and ranging from 119,722 to 137,506 reads per sample) and 15,906,512 16S rRNA raw sequences (averaging 122,357 and ranging from 72,930 to 137,843 reads per sample) from 125 samples. After the short sequences and chimeric sequences were removed, 16,190,548 ITS high-quality sequences (averaging 124,542 and ranging from 119,681 to 137,481 reads per sample) and 15,868,802 16S rRNA high-quality sequences (averaging 122,067 and ranging from 72,399 to 137,767 reads per sample) were obtained for analysis (Supplementary Tables S1, S2). The observed species (sobs) curve and the rank abundance distribution curve were plotted to reflect the microbial diversity and species abundance in the samples (Supplementary Figures S1A–S1D). The number of high-quality sequences was sufficient to reflect the vast majority of microbial information in the sample. At the 97% sequence similarity level, the fungal sequences clustered into 16,094 OTUs, and the bacterial sequences clustered into 53,671 OTUs (Supplementary Tables S3, S4). The bacterial richness was significantly higher than the fungal richness.

### 3.2 Species Composition Analysis

**FIGURE 1:**
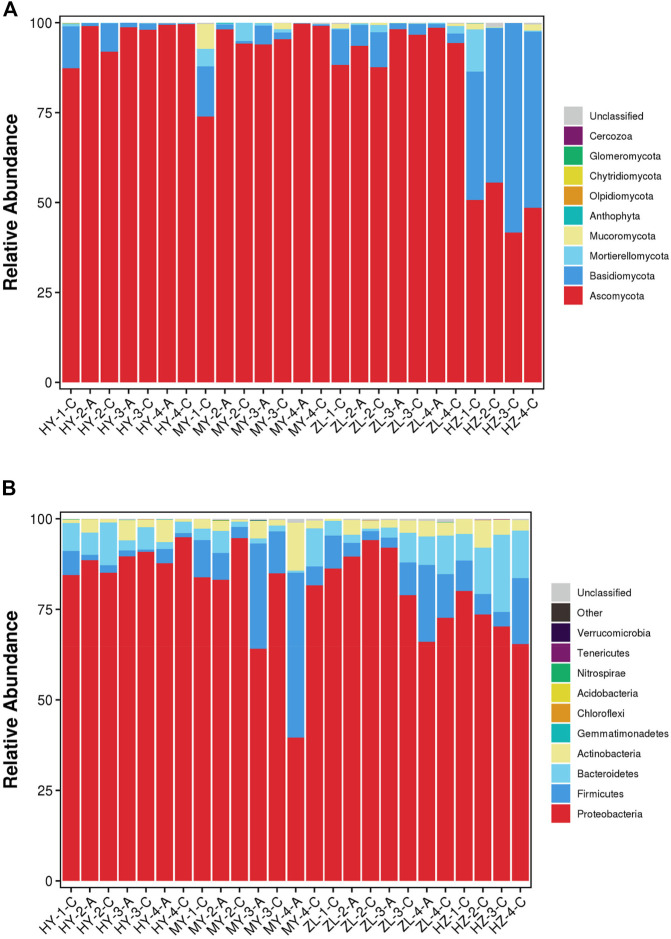
The stacked bar plot of the community composition and relative abundances of representative fungal **(A)** and bacteria **(B)** on the phylum level of communities detected in the samples of diseased potato tubers.

At the phylum level, *Ascomycota* was the dominant fungal phylum, but there were regional differences in the abundance of each sample. In the HY, MY and ZL samples, the relative abundance of *Ascomycota* was >73%, while in HZ, except for *Ascomycota*, which accounted for 41–55%, *Basidiomycota* was also the dominant fungal phylum, accounting for 42–58% (Figure 1A). Regarding bacteria, *Proteobacteria* was the dominant bacterial phylum, with an average abundance of 77.76%. In addition, the proportion of *Firmicutes* and *Bacteroidetes* was relatively high in individual samples, with averages of 8.7 and 5.9%, respectively (Figure 1B).

**FIGURE 2:**
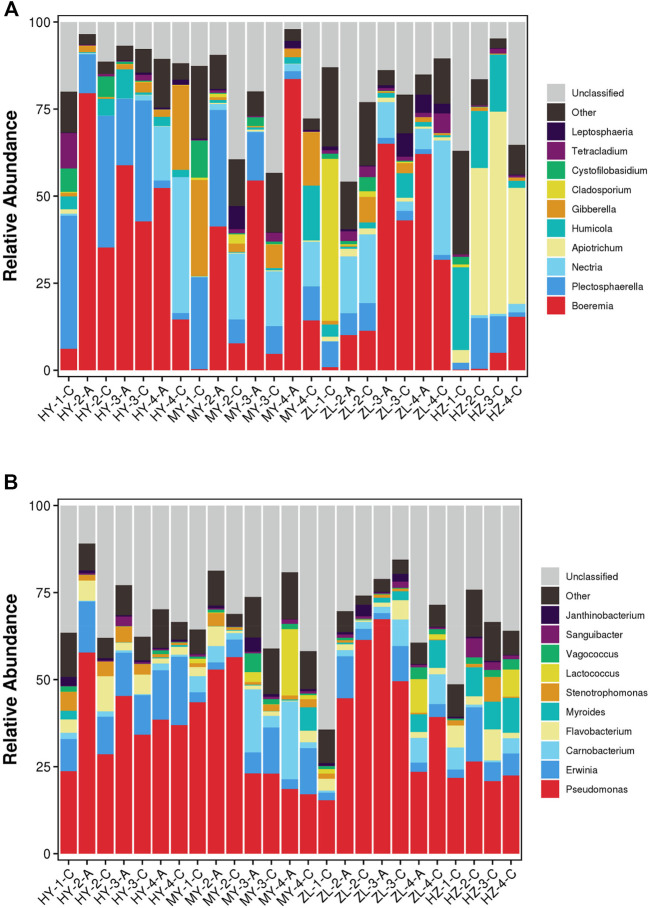
The stacked bar plot of the community composition and relative abundances of representative fungal **(A)** and bacteria **(B)** on the genus level of communities detected in the samples of diseased potato tubers.

At the genus level, the top ten dominant fungi were *Boeremia*, *Plectosphaerella*, *Nectria*, *Apiotrichum*, *Humicola*, *Gibberella*, *Cladosporium*, *Cystofilobasidium*, *Tetracladium*, and *Leptosphaeria*, but the abundance was different in different regions. *Boeremia* changed greatly in each sample, and it was higher in treatment Group “A” (average abundance was 56%) than that in control Group “C” (average abundance was 14%). In contrast, *Plectosphaerella* and *Nectria* in Group “C” (average abundance was 13 and 9% respectively) were higher than those in Group “A” (average abundance was 10 and 5% respectively), and other genera with high abundance, such as *Gibberella* and *Cladosporium*, also appeared in Group “C.” In addition, with the prolongation of storage time, in Group “A,” *Boeremia* in HY decreased continuously but increased continuously in MY and decreased after increasing in ZL. However, in Group “C,” *Boeremia* first increased and then decreased in HY and ZL but continued to increase in MY and HZ. In addition, *Apiotrichum* and *Humicola* were the dominant genera in HZ, with average abundances of 34.3 and 14.5%, respectively, in which *Apiotrichum* increased at first and then decreased with the change in storage time, while *Humicola* decreased continuously (Figure 2A, Supplementary Figure S2A). The top ten dominant bacteria were *Pseudomonas*, *Erwinia*, *Carnobacterium*, *Flavobacterium*, *Myroides*, *Stenotrophomonas*, *Lactococcus*, *Vagococcus*, *Sanguibacter*, and *Janthinobacterium*. *Pseudomonas* was the absolutely dominant genus of bacteria with an average abundance of 35%, and its dynamics in each sample showed that the abundance of *Pseudomonas* in Group “A” (average abundance was 47%) was higher than that in Group “C” (average abundance was 30%) in the HY area. However, in ZL, its abundance in Group “C” was higher in the second and fourth periods and lower in the third period. Furthermore, with the change in storage time, in Group “A,” *Pseudomonas* decreased gradually in HY and MY, increased at first and then decreased in ZL. In Group “C,” however, it increased gradually in HY and increased at first and then decreased in MY, ZL and HY, but the highest abundance appeared in the second period (Figure 2B, Supplementary Figure S2B).

### 3.3 Species Difference Analysis and Biomarkers

**FIGURE 3:**
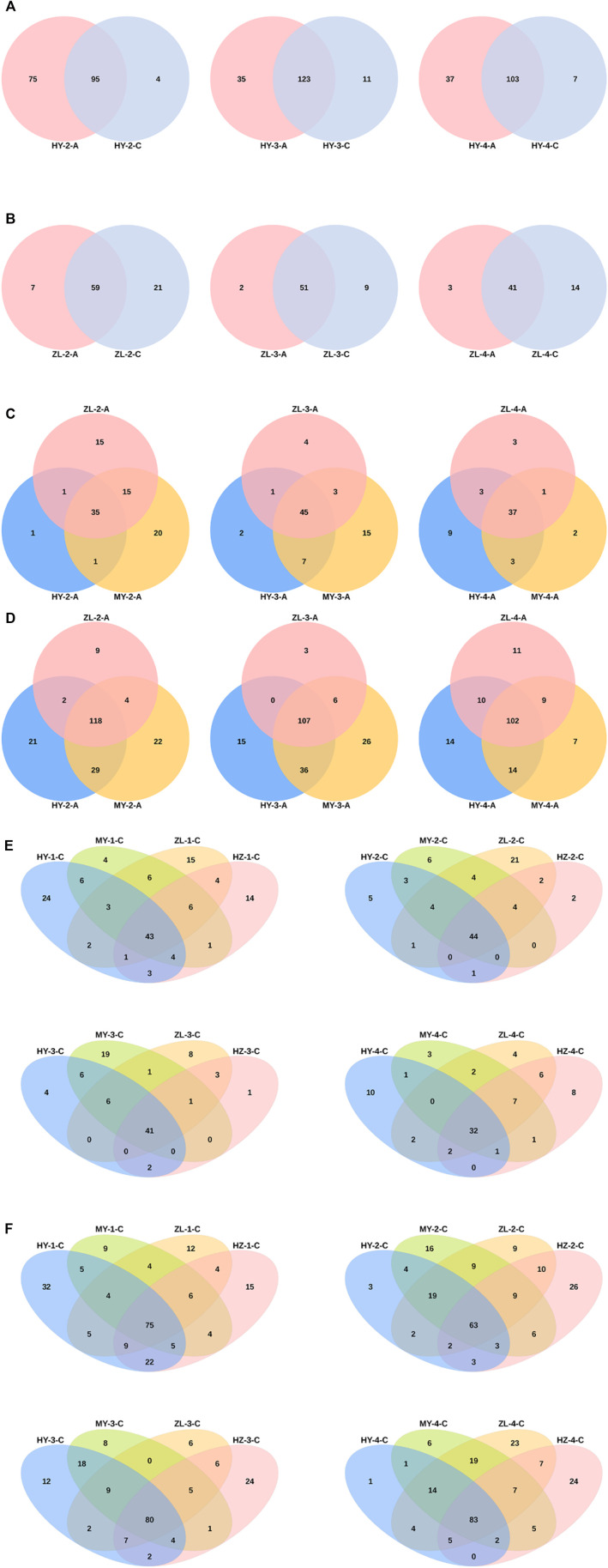
Venn Analysis of different species between the treatment group “A” and control group “C” [**(A)** for fungi and **(B)** for bacteria], among regions in group “A” [**(C)** for fungi and **(D)** for bacteria] and in group “C” [**(E)** for fungi and **(F)** for bacteria] at the genus level.

Venn analysis of different species showed that treatment Group “A” had more unusual species of fungi and fewer bacteria than control Group “C” at the genus level in most of the samples (Figures 3A,B, Supplementary Figures S3A, S3B). In Group “A,” the numbers of common genera of fungi in the three periods were 35, 45 and 37, and the numbers of bacteria were 118, 107 and 102. Furthermore, in the first and second periods, most of the endemic species were included in MY, but in the fourth period, they were included in HY (Figures 3C,D). For samples of Group “C,” the numbers of common fungal species in HY, MY, ZL and HZ at different periods were 43, 44, 41, and 32, respectively, less than those of the bacteria, which were 75, 63, 80, and 83. Among them, more unusual fungal species were enriched in HY, and bacterial species were enriched in HZ (Figures 3E,F). Furthermore, in the comparison of different storage periods, the samples in Group “A” had the most endemic species in the second period (Supplementary Figures S3C, S3D), while in Group “C,” there were more endemic species in the first and fourth periods (Supplementary Figures S3E, S3F).

**FIGURE 4:**
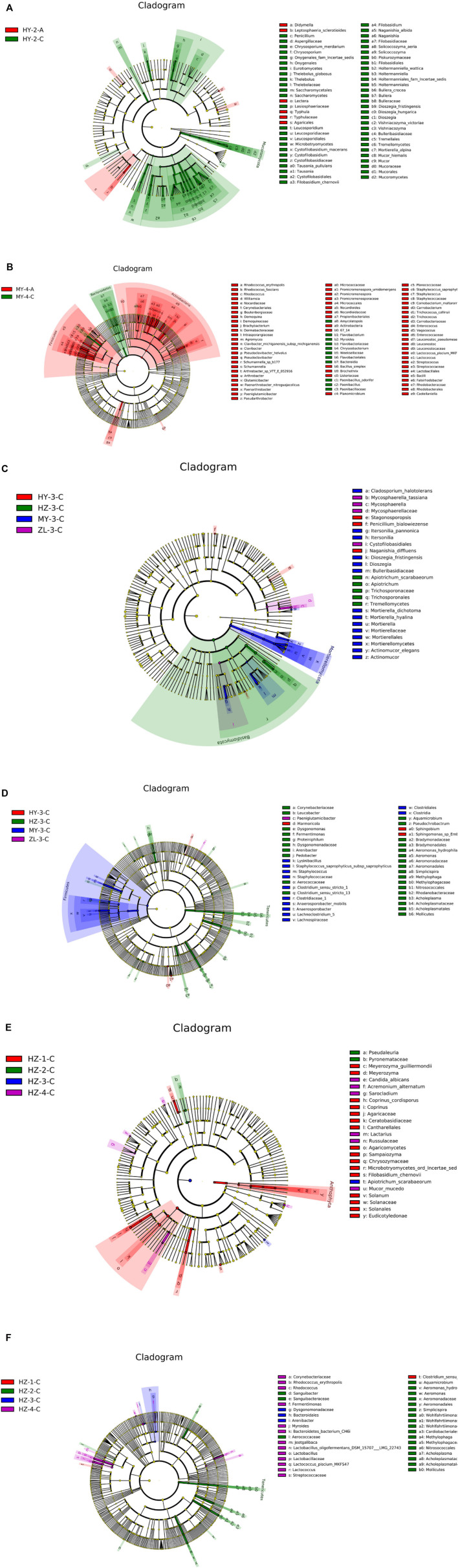
The sample with the most biomarkers between Group “A” and Group “C” [**(A)** for fungi and **(B)** for bacteria], among storage periods [**(C)** for fungi and **(D)** for bacteria] and different regions [**(E)** for fungi and **(F)** for bacteria].

The biomarker features in each group were screened by LEfSe, and the results showed that the most differential species of fungi between Groups “A” and Groups “C” appeared in the second period in HY, and the samples of Group “C” had more biomarker species (Figure 4A). The most differential species of bacteria were found in the fourth period in MY, and there were more biomarker species in the samples of Group “A” (Figure 4B). Furthermore, in the analysis of biomarker features in different storage periods, most biomarkers of fungi and bacteria were present in the third period, among which fungi had the most biomarkers in HZ and bacteria had the most biomarkers in MY (Figures 4C,D). However, in the comparison among different regions at the same storage time, the number of biomarkers in HZ was the largest, among which fungi in the first period and bacteria in the second period showed the largest diversity (Figures 4E,F).

**FIGURE 5:**
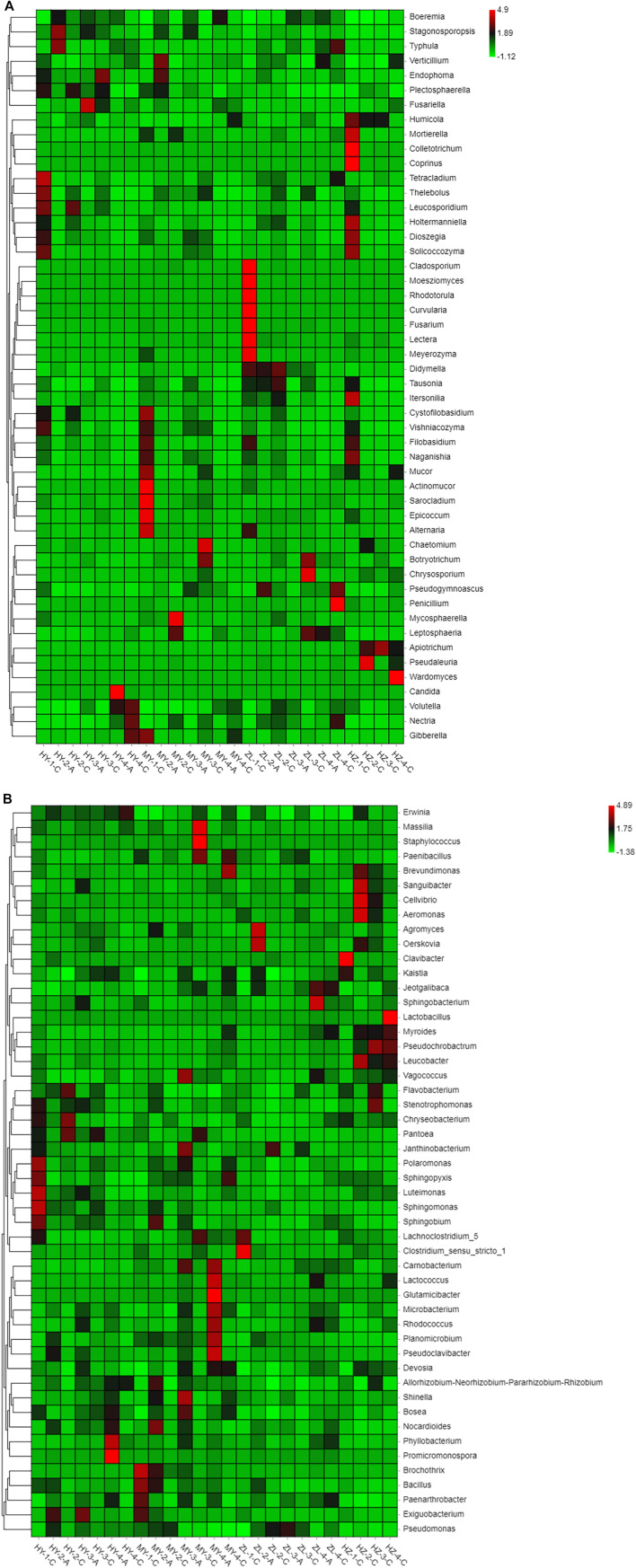
The relative abundances heatmap of the top 50 genera of fungi **(A)** and bacteria **(B)**.

We further utilized the relative abundances of the top 50 genera to generate a heatmap, and the heatmap shows that potato pathogens, such as those belonging to the genera *Fusarium*, *Alternaria*, and *Colletotrichum,* which inflict serious damage during potato storage, are among the top 50 genera in terms of relative abundance. *Fusarium* was mainly present in group ZL-1-C, while *Alternaria* was mainly present in groups MY-1-C and ZL-1-C, and *Colletotrichum* was mainly present in group HZ-1-C. However, some potential antagonist fungi, such as *Mucor* and *Penicillium,* that have antagonistic effects on plant pathogens had higher abundance in groups MY-1-C and ZL-4-C (Figure 5A). For bacteria, the pathogen of potato soft rot belonging to *Erwinia* was enriched in the samples HY-4-C, HZ-2-C, HY-2-A, MY-3-C, and MY4-C, etc., while potentially antagonistic bacteria such as *Bacillus*, *Pseudomonas*, *Pantoea* and *Exiguobacterium* were concentrated in groups MY-1-C, MY2-A, ZL-3-A, ZL-2-C, HY-2-C, and HY-3-A (Figure 5B). In general, pathogenic fungi and bacteria were both concentrated in Group “C,” while antagonistic fungi were also concentrated in Group “C,” but antagonistic bacteria were distributed to different degrees in Group “A” and Group “C.”

### 3.4 Microbial Diversity Analysis of Diseased Potato Tuber Samples

#### 3.4.1 Alpha Diversity Analysis

**FIGURE 6:**
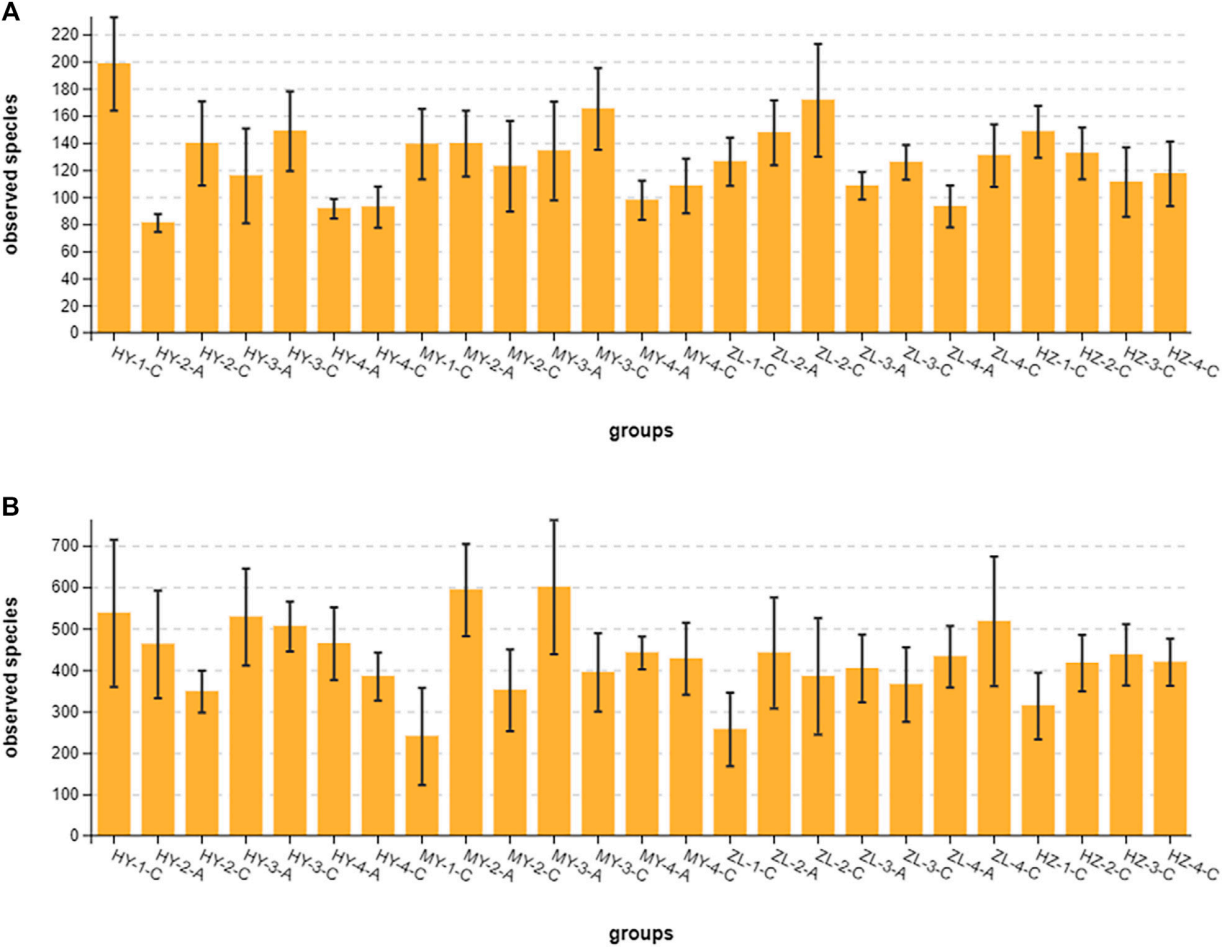
The observed-species index of Alpha diversity of fungi **(A)** and bacteria **(B)**.

The coverage rate of the sequencing depth index of each sample was more than 0.99, indicating that the amount of sequencing was sufficient to cover the colony composition of the samples. The richness and diversity of the microbial community were analyzed by the alpha diversity index: Chao1, ACE, Shannon, Simpson, Good’s coverage, and Pielou’s evenness index (Supplementary Tables S5, S6). The results showed that the fungal diversity index in control Group “C” was higher than that in treatment group “A” in all samples except MY-2-C (Figure 6A). There were significant differences in the fungal observed-species index (richness) between Groups “A” and Groups “C” in the second period in HY and in the third and fourth periods in ZL (*p* < 0.05) (Table 2). For the storage time, there were significant differences among each period of Group “C” in HY and all samples of group “A” in each period. Among regions, however, there were significant differences in the first and third periods in Group “C” and in the second period in Group “A” (Table 2). The changes in the observed species index in Group “A” first increased and then decreased in the HY area, and the peak appeared in the second period, while it decreased continuously in MY and ZL, and the peak value was in the first period. In Group “C,” the highest abundance was in the first stage in HY and HZ and then decreased gradually, while it was present in the third and second periods in MY and ZL, respectively, which increased at first and then decreased. With the extension of the cellular period, the fungal diversity of all samples decreased to its lowest value in the fourth period (Figure 6A).

**TABLE 2:** The test of observed-species index among groups of Alpha diversity of fungi and bacteria.

Test	Group	Fungal		Bacterial	
		Pvalue	Significant	Pvalue	Significant
Welch’s *t*-test	HY-2-A-VS-HY-2-C	0.01	*	0.12	
	HY-3-A-VS-HY-3-C	0.15		0.71	
	HY-4-A-VS-HY-4-C	0.88		0.14	
	MY-2-A-VS-MY-2-C	0.39		0.01	**
	MY-3-A-VS-MY-3-C	0.18		0.05	*
	MY-4-A-VS-MY-4-C	0.37		0.74	
	ZL-2-A-VS-ZL-2-C	0.30		0.53	
	ZL-3-A-VS-ZL-3-C	0.05	*	0.50	
	ZL-4-A-VS-ZL-4-C	0.02	*	0.32	
Kruskal–Wallis test	HY-1-C-VS-HY-2-C-VS-HY-3-C-VS-HY-4-C	0.01	**	0.01	**
	MY-1-C-VS-MY-2-C-VS-MY-3-C-VS-MY-4-C	0.05		0.11	
	ZL-1-C-VS-ZL-2-C-VS-ZL-3-C-VS-ZL-4-C	0.10		0.03	*
	HZ-1-C-VS-HZ-2-C-VS-HZ-3-C-VS-HZ-4-C	0.09		0.10	
	HY-1-C-VS-MY-1-C-VS-ZL-1-C-VS-HZ-1-C	0.02	*	0.02	*
	HY-2-C-VS-MY-2-C-VS-ZL-2-C-VS-HZ-2-C	0.15		0.43	
	HY-3-C-VS-MY-3-C-VS-ZL-3-C-VS-HZ-3-C	0.04	*	0.10	
	HY-4-C-VS-MY-4-C-VS-ZL-4-C-VS-HZ-4-C	0.09		0.42	
	HY-2-A-VS-HY-3-A-VS-HY-4-A	0.02	*	0.62	
	MY-2-A-VS-MY-3-A-VS-MY-4-A	0.04	*	0.11	
	ZL-2-A-VS-ZL-3-A-VS-ZL-4-A	0.01	*	0.87	
	HY-2-A-VS-MY-2-A-VS-ZL-2-A	0.01	**	0.21	
	HY-3-A-VS-MY-3-A-VS-ZL-3-A	0.25		0.10	
	HY-4-A-VS-MY-4-A-VS-ZL-4-A	0.54		0.78	

For bacteria, the observed species index of all samples in treatment Group “A” was higher than that in control Group “C” except ZL-4-A (Figure 6B). There were significant differences in the bacterial diversity index between Group “A” and Group “C” in the second and third periods in MY (*p* < 0.05) (Table 2). There were also significant differences among the four periods of Group “C” in HY and ZL. Furthermore, there were significant differences in the first period among regions in Group “C” (Table 2). In Group “A,” the diversity index first increased and then decreased in HY and MY, while it first decreased and then increased in ZL. In Group “C,” the changes of the diversity index in different regions were diverse, decreasing at first and then increasing in the HY area, decreasing continuously in MY, increasing continuously in ZL, and increasing at first and then decreasing in HZ. The period with the lowest diversity index appeared was the first period in the HY area and the fourth period in other areas (Figure 6B).

#### 3.4.2 Beta Diversity Analysis

**FIGURE 7:**
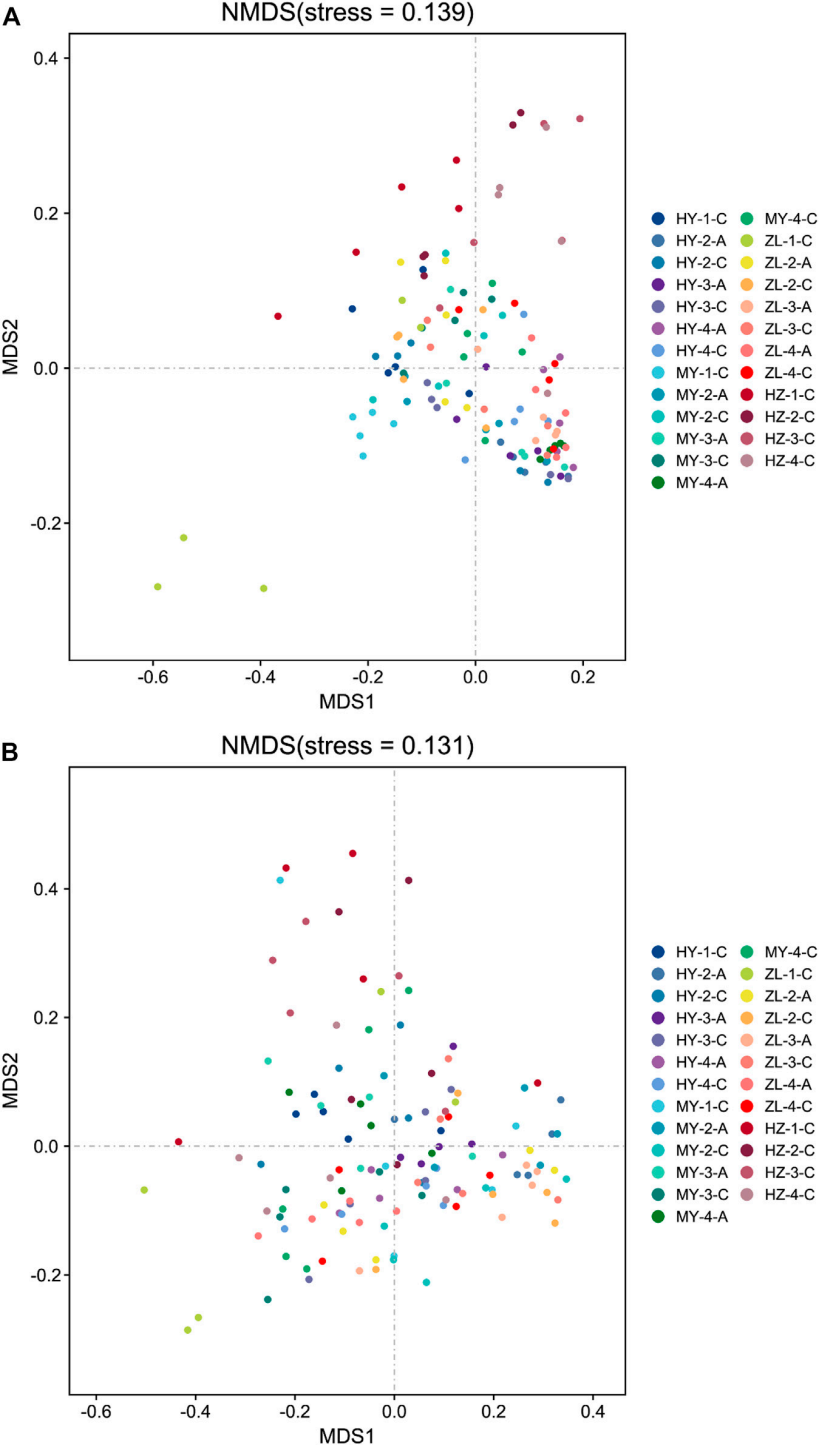
The non-metric multidimensional scaling (NMDS) of fungal **(A)** and bacterial **(B)** community compositions.

The differences in the fungal and bacterial community compositions were compared using NMDS based on the Bray–Curtis distance. The results showed that samples aggregated in the NMDS analysis diagram of fungal community compositions, while they were dispersed in bacterial community compositions (Figure 7). The fungal community compositions between treatment Group “A” and control Group “C” were similar in ZL and HZ (Figure 7A). The results of the ANOSIM test showed that the Bray–Curtis distances were significantly different between treatment Group “A” and control Group “C” for all samples in MY and the samples from the fourth period in HY (*p* < 0.05) (Table 3). In the comparison among storage periods, only the samples of group “A” in HY were similar in each period, while there were significant differences in all of the samples in other areas during the storage period. There were also significant differences among storage periods in all samples except HY-A. Among the regions, however, there were significant differences among regions only in the second period (Table 3).

**TABLE 3:** The Anosim test of fungal and bacterial community compositions.

Groups	Fungal			Bacterial		
	Rvalue	Pvalue	Significant	Rvalue	Pvalue	Significant
HY-2-A-vs-HY-2-C	0.20	0.08		0.36	0.04	*
HY-3-A-vs-HY-3-C	0.00	0.32		0.04	0.34	
HY-4-A-vs-HY-4-C	0.49	0.01	*	−0.10	0.69	
MY-2-A-vs-MY-2-C	0.36	0.01	**	0.06	0.26	
MY-3-A-vs-MY-3-C	0.56	0.01	**	0.11	0.23	
MY-4-A-vs-MY-4-C	0.74	0.01	*	0.72	0.01	*
ZL-2-A-vs-ZL-2-C	−0.12	0.73		0.00	0.36	
ZL-3-A-vs-ZL-3-C	0.03	0.31		0.13	0.14	
ZL-4-A-vs-ZL-4-C	0.18	0.11		0.00	0.47	
HY-1-C-vs-HY-2-C-vs-HY-3-C-vs-HY-4-C	0.43	0.00	**	0.10	0.11	
MY-1-C-vs-MY-2-C-vs-MY-3-C-vs-MY-4-C	0.33	0.00	**	0.25	0.01	*
ZL-1-C-vs-ZL-2-C-vs-ZL-3-C-vs-ZL-4-C	0.35	0.00	**	0.21	0.00	**
HZ-1-C-vs-HZ-2-C-vs-HZ-3-C-vs-HZ-4-C	0.17	0.05	*	0.19	0.01	*
HY-1-C-vs-MY-1-C-vs-ZL-1-C-vs-HZ-1-C	0.34	0.00	**	0.09	0.09	
HY-2-C-vs-MY-2-C-vs-ZL-2-C-vs-HZ-2-C	0.44	0.00	**	0.37	0.00	**
HY-3-C-vs-MY-3-C-vs-ZL-3-C-vs-HZ-3-C	0.49	0.00	**	0.25	0.00	**
HY-4-C-vs-MY-4-C-vs-ZL-4-C-vs-HZ-4-C	0.42	0.00	**	0.30	0.00	**
HY-2-A-vs-HY-3-A-vs-HY-4-A	0.12	0.06		0.26	0.02	*
MY-2-A-vs-MY-3-A-vs-MY-4-A	0.25	0.02	*	0.42	0.01	**
ZL-2-A-vs-ZL-3-A-vs-ZL-4-A	0.25	0.02	*	0.37	0.00	**
HY-2-A-vs-MY-2-A-vs-ZL-2-A	0.40	0.00	**	0.00	0.47	
HY-3-A-vs-MY-3-A-vs-ZL-3-A	0.08	0.15		0.49	0.00	**
HY-4-A-vs-MY-4-A-vs-ZL-4-A	0.11	0.08		0.50	0.00	**

Regarding bacterial community compositions, although they were relatively scattered, they also showed a similar pattern between treatment Group “A” and control Group “C” in ZL and HZ (Figure 7B). There were significant differences between Group “A” and Group “C” in the second period in HY and the fourth period in MY (*p* < 0.05) (Table 3). In the comparison of different storage times, in Group “C,” only the samples were similar in the four periods in HY, while there were significant differences between storage periods in MY, ZL and HZ. All of the samples in Group “A” were significantly different in different periods (Table 3). In addition, in the comparison of different regions, in Group “C,” the samples from four regions were similar only in the first period, and there were significant differences in the second, third and fourth periods. However, in Group “A,” samples from different regions were only similar in the second period, and there were significant differences in the third and fourth periods (Table 3). The results showed that the community compositions of fungi and bacteria were affected by different treatments, ecological regions, and storage periods.

### 3.5 Analysis of the Plant Pathogenicity in the Microbial Communities in the Samples of Diseased Potatoes

**FIGURE 8:**
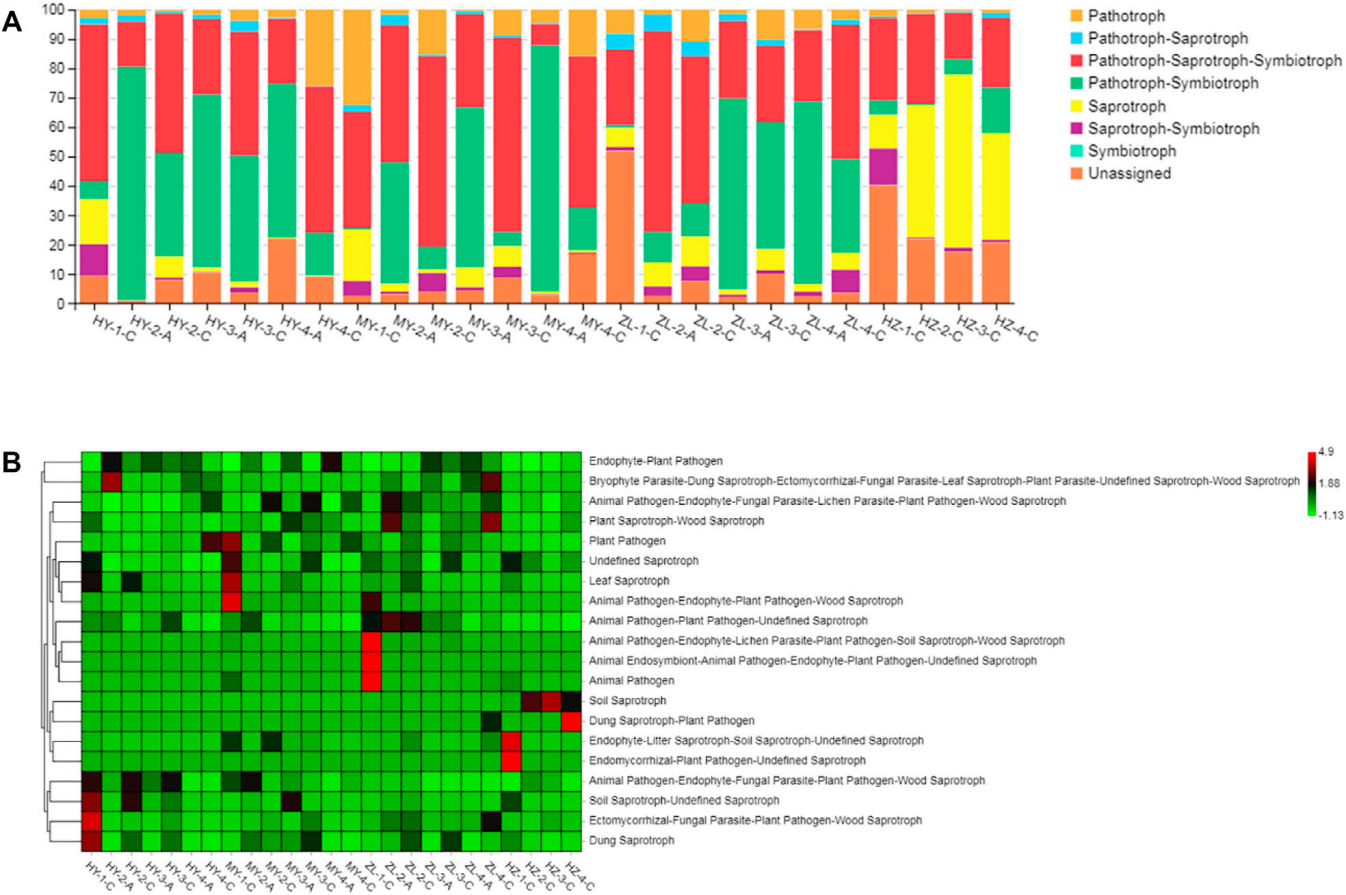
The main nutritional types of fungi of diseased potato tubers at the level of trophic **(A)** and guild **(B)** classification.

**FIGURE 9:**
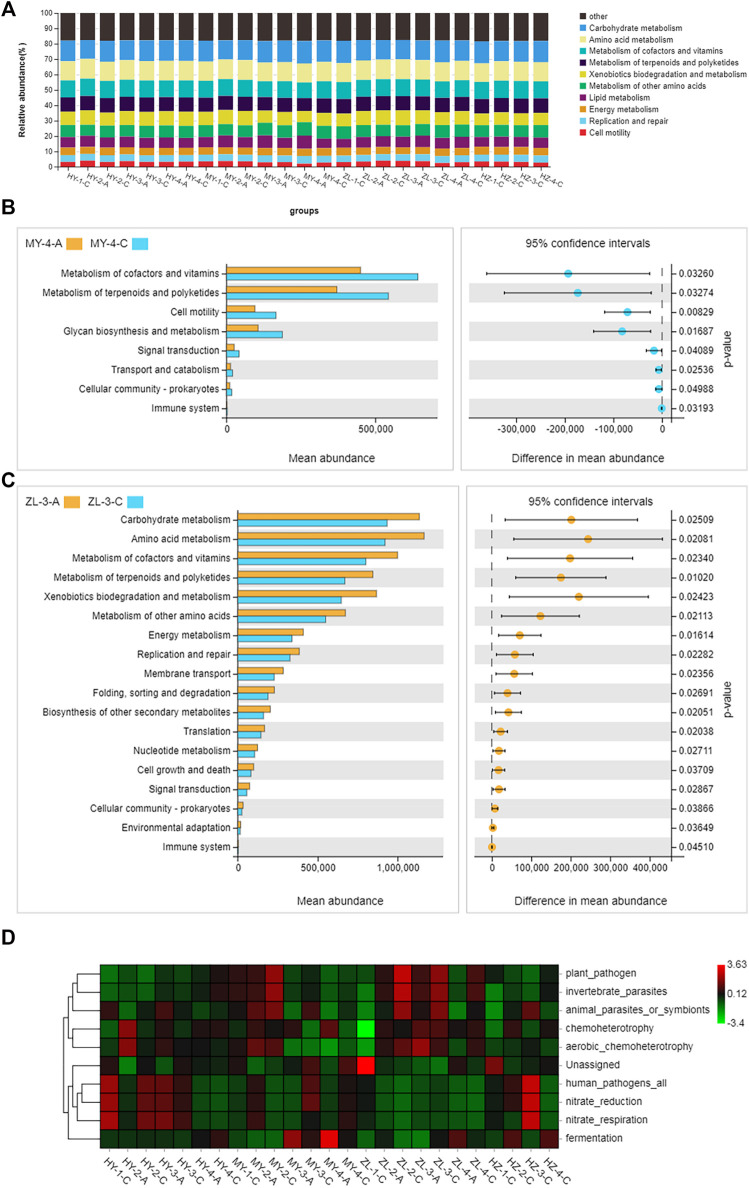
The main metabolic pathways of bacteria of diseased potato tubers **(A)** and test between group “A” and group “C” **(B,C)**, and the function of top10 **(D)**.

The plant pathogenic fungi that occurred in the diseased potato samples were inferred using FUNGuild. At the level of trophic classification, the main nutritional types were Pathotroph-Saprotroph-Symbiotroph, Pathotroph-Symbiotroph and Saprotroph. However, their abundance in each sample was different, in which Pathotroph-Saprotroph-Symbiotroph type fungi in Group “C” (average abundance was 41%) were obviously higher than those in Group “A” (average abundance was 29%), while Pathotroph-Symbiotroph fungi in Group “A” (average abundance was 56%) were higher than those in Group “C” (average abundance was 14%). Pathotroph and Saprotroph were also higher in Group “C” (average abundance was 8 and 14%, respectively) than those in Group “A” (average abundance was 2.5 and 2.8%, respectively), and in particular, Saprotroph in HY samples accounted for 12–59% (Figure 8A). When further subdivided into 60 guilds (Supplementary Table S7), we found that some of the fungal species were plant pathogens by analyzing the predicted results, and they were also more abundant in Group “C” of HY, MY and ZL, while the fungal species of saprotrophs were more abundant in HZ, such as soil saprotrophs, dung saprotrophs, and plant saprotrophs (Figure 8B). The KEGG pathway analysis of bacteria was inferred using PICRUSt, and the main metabolic pathways were carbohydrate metabolism, amino acid metabolism and metabolism of cofactors and vitamins (Figure 9A). Seven pathways were more abundant in Group “C” in MY-4, while seventeen pathways were higher in Group “A” in ZL-3 (Figures 9B,C). Notably, plant pathogenic bacteria were enriched in most of the samples and significantly enriched in MY-2-C, ZL-2-C, and ZL-3-C (Figure 9D).

### 3.6 Co-Occurrence Network Construction and Analysis

**FIGURE 10:**
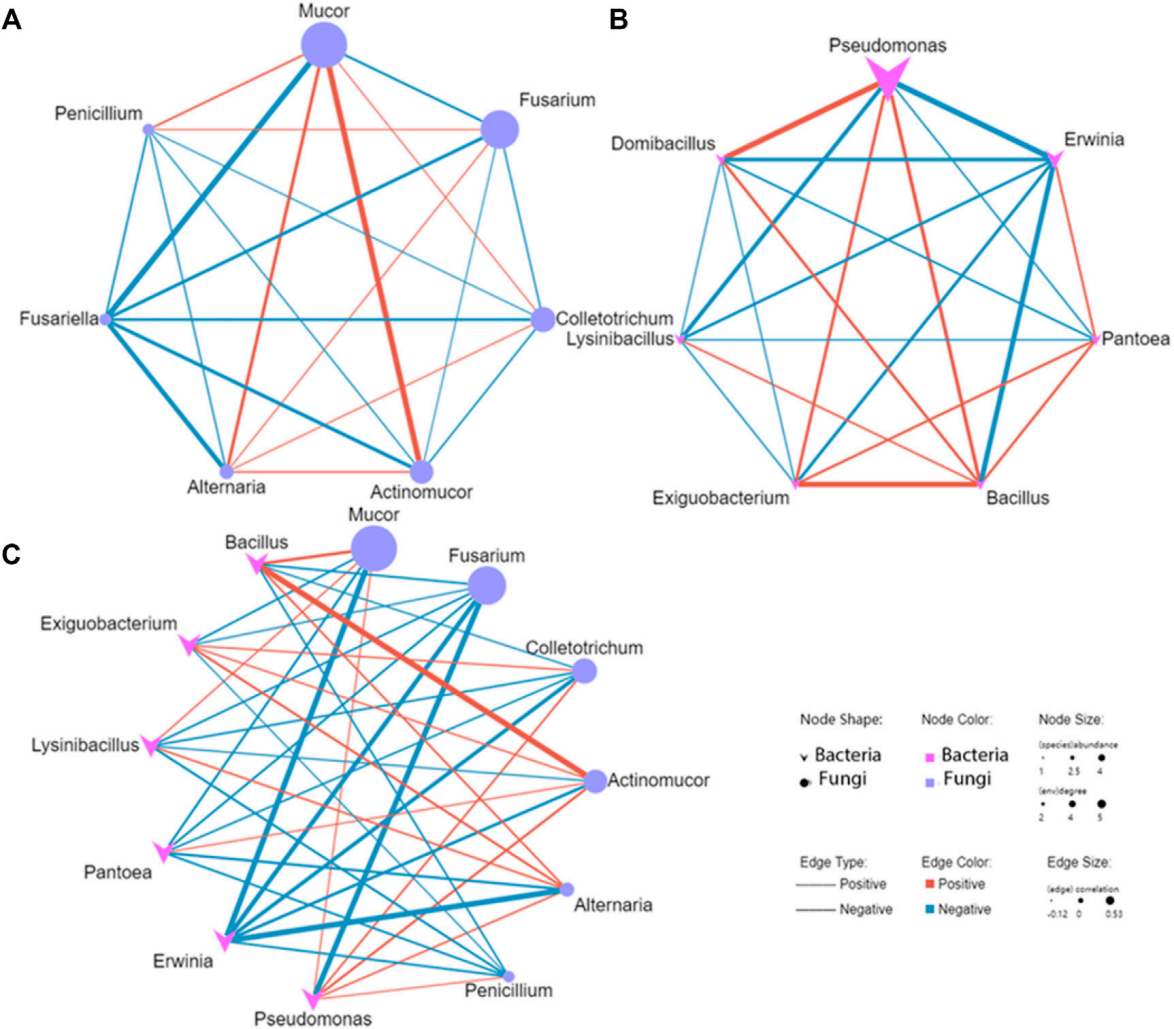
The Co-occurrence network between Pathogens and antagonistic microbe of diseased potato tuber [fungi **(A)**, bacteria **(B),** fungi and bacteria **(C)**].

We conducted a co-occurrence network analysis of the concerned species to evaluate the significant correlation (*p* < 0.01) of pathogens and antagonistic microbe in diseased potato tubers (Figure 10). In the correlation analysis of the interaction network between pathogenic fungi *Fusarium*, *Alternaria*, and *Colletotrichum* and antagonistic fungi *Mucor* and *Penicillium*, there was a negative correlation between *Fusarium* and *Mucor*, while *Alternaria* and *Colletotrichum* negatively correlated with *Penicillium*. In the analysis of pathogenic fungi, *Alternaria* positively correlated with *Fusarium* and *Colletotrichum*, while there was a negative correlation between *Fusarium* and *Colletotrichum*, for the antagonistic fungi, however, there was a positive correlation between *Mucor* and *Penicillium* (Figure 10A). In the interaction between pathogenic bacteria *Erwinia* and antagonistic bacteria *Bacillus*, *Pseudomonas*, *Pantoea* and *Exiguobacterium*, *Erwinia* was negatively correlated with all the antagonistic bacteria except *Pantoea*, while there was a positive correlation between *Bacillus* and other antagonistic bacteria (Figure 10B). In the interaction between fungi and bacteria, the pathogenic fungus *Fusarium* was negatively correlated with all selected bacteria, while the pathogenic bacteria *Erwinia* was negatively correlated with all selected fungi (Figure 10C). Specifically, pathogenic fungus *Fusarium* and *Colletotrichum* where negatively correlated with antagonistic bacteria *Bacillus*. While antagonistic fungi *Mucor* and *Actinomucor* were positively correlated with *Bacillus*. Pathogenic bacteria *Erwinia* were negatively correlated with antagonistic fungi, and strongly negatively correlated with *Mucor* and *Alternaria*. The results showed that there was a complex interaction between pathogens and antagonists in diseased potato tubers, especially the negative correlation between pathogens and antagonistic microbe, which revealed the interaction between microorganisms in diseased potato tubers.

## Discussion

At present, there are many studies on the microbial community during food storage that have utilized high-throughput sequencing. For example, in *Antarctic krill*, the dominant bacteria causing corruption during storage were *Colchrobactersp*, and the dominant fungi were unclassified yeasts (Wang et al., 2021). Regarding potato storage, there is only one study on the microbial diversity of fresh potato slices, in which the dominant flora of fresh-cut potatoes was *Proteobacteria* and *Firmicutes* at different storage times. However, there have been few studies on the microbial diversity of diseased potato tubers during storage. In this study, healthy potatoes were dried before entering the cellar, and then the microbial community of diseased potato tubers was analyzed by high-throughput sequencing and compared in different ecological regions and storage periods. The results showed that the bacterial communities were richer than fungal communities during storage, which was consistent with the results of dry red wine bream (*Lutianus erythropterus*) (Deng et al., 2020). *Ascomycota* and *Basidiomycota* were the dominant phyla of diseased potato tubers with the abundance of 73–99% and 42–58%, respectively, which were also dominant in the potato rhizosphere with the abundance of 63 and 15%, respectively, but the dominant genera were different. *Peyronellaea* was the dominant genus in the potato rhizosphere, while the dominant genus in diseased potato tubers was *Boeremia*, which is an endophytic fungus of potato (Zimudzi et al., 2018; Yao et al., 2020). In addition, the abundance of *Boeremia* in treatment Group “A” (average abundance was 56%) was higher than that in control Group “C” (average abundance was 14%), but the opposite phenomenon was reflected in the genera that also have a higher relative abundance, such as *Plectosphaerella*, *Nectria*, *Gibberella*, and *Cladosporium*, the abundance of which was higher in Group “C” (average abundance was 13, 9, 5, and 3%, respectively) than that in treatment Group “A” (average abundance was 10, 5, 1, and 0.2%, respectively), and they are important pathogenic fungi of potato, wheat, corn and other plants (Etebu and Osborn, 2010; Jashni et al., 2019; Alam et al., 2021). The dominant bacterial genus of diseased potatoes during storage is *Pseudomonas* with an average abundance of 35%, and it was also reported that the relative abundance of *Pseudomonas* was the highest in mutton chop rolls stored for 8 days with the highest content reached 95% (Zhang et al., 2020). Some species of *Pseudomonas* are pathogenic bacteria of potato, while some can be used as antagonistic strains to inhibit potato pathogens (Simon et al., 2018; Terletskiy and Lazarev, 2019; Tomar et al., 2019; Zlatković et al., 2021). Furthermore, in the analysis of species level, *Pseudomonas syringae pv. tomato* was the dominant species and was one of the common pathogenic bacteria in plants (Primo et al., 2019; Elsharkawy et al., 2021; Yengkhom and Supriyo, 2021). *Erwinia* was also the dominant bacterium with an average abundance of 8% in diseased potatoes in our study, as the reported pathogen of potato soft rot (Makhlouf and Abdeen, 2014; Fujimoto et al., 2018; Hua et al., 2020), so these two dominant bacteria, *Pseudomonas* and *Erwinia,* may be the pathogenic bacteria causing potato diseases in our study. In addition, bacteria of the genera *Flavobacteriaceae* and *Carnobacteriaceae*, which also existed in diseased potatoes, have been reported to be the main components leading to meat deterioration during meat storage(Zhao et al., 2015). In the analysis of indicator species, *Fusarium*, *Alternaria* and *Colletotrichum*, which are the important pathogenic fungi of potatoes, were mostly enriched in Group “C,” while the potentially antagonistic fungi *Mucor* and *Penicillium* (Mardani and Hadiwiyono 2018; Teixeira et al., 2012) were also present in Group “C,” but antagonistic bacteria, such as *Bacillus*, *Pantoea*, and *Exiguobacterium* (Abo-Elyousr and Hassan, 2021; Cavanaugh et al., 2021; Fernandes et al., 2021), were distributed to varying degrees in Group “A” and Group “C.” Further functional predictive analysis confirmed that Pathotroph-Saprotroph-Symbiotroph, Pathotroph-Symbiotroph and Saprotroph were the main nutritional types of fungi in diseased potato tubers. In addition, the Pathotroph-Saprotroph-Symbiotroph type in Group “C” (average abundance was 41%) was significantly higher than that in Group “A” (average abundance was 29%), while Pathotroph-Symbiotroph in Group “A” (average abundance was 56%) was higher than that in Group “C” (average abundance was 14%), furthermore, the types of Pathotroph and Saprotroph were also higher in Group “C”. Further analysis of the functional groups of the fungal community revealed that the plant–pathogens were also abundant in Group “C” of HY, MY and ZL, while the fungal species of Saprotroph were more abundant in HZ, such as soil Saprotroph, dung Saprotroph and plant Saprotroph, etc. This result is consistent with reports of *Coptis Chinensis* in which the plant pathogens in the rhizosphere of diseased plants were significantly higher than those of healthy plants (Liao et al., 2020). Therefore, these fungi may be the main pathogens of potato and the treatment were significantly reduced the distribution of pathogens.

In terms of dynamic changes in microbes during storage, there were fluctuations in different regions. For example, the dominant genera *Boeremia* and *Pseudomonas* showed continuous increases, continuous decreases or first increases and then decreases in different regions. The pattern of first increasing and then decreasing also appeared with the change in mold counts during japonica rice storage (Shi et al., 2021). Similar changes were observed in the microbial diversity of diseased potato tubers. The fungal *α*-diversity in control Group “C” was more abundant than that in treatment Group “A,” but the bacterial diversity was lower. This is opposite to the results of the study on *C. chinensis*, in which the α diversity of the rhizosphere fungi of the diseased plants was significantly higher than that of the healthy plants, and the α diversity of the bacteria in the endosphere of the diseased plants was significantly higher than that of the healthy plants (Liao et al., 2020). This may be because the method of selection and drying could reduce the fungi brought into the cellar and reproduce in the cellar. Compared with the increase in cellar time, the fungal *α* diversity index showed a downward trend. There were significant differences in fungal *α* diversity among the four periods in HY of Group “C” and all of the samples of Group “A,” while there were significant differences in the bacterial *α* diversity among different periods in HY and ZL. This result indicates that with the extension of time, the disease tissue underwent severe rotting, resulting in a decrease in fungal diversity and an increase in bacterial diversity.

There were large differences in the microbial community structures of samples from different geographical regions, especially the fungi *Boeremia*, *Plectosphaerella* and *Nectria,* which were the dominant genera in HY, MY and ZL, while in HY, the dominant genera were *Apiotrichum* and *Humicola*. For bacteria, except for *Pseudomonas*, which was the absolute dominant bacterium in all samples, *Carnobacterium* was mainly distributed in the MY and ZL samples, while *Myroides* was more abundant in the HZ samples. In addition, in the analysis of *α* diversity, there were significant differences among different regions of fungal diversity in Group “C” at the first and third periods and significant differences in Group “A” at the second period. The *α* diversity of bacteria was similar only in the first period of Group “C,” and there were significant differences among the four regions. The *β* diversity of fungi of all samples in control Group “C” was extremely significant (*p* < 0.01), while the bacterial *β* diversity in the four regions was significantly different in the second, third and fourth periods. In Group “A,” the fungal *β* diversity showed significant differences only in the second period; in contrast, the bacterial β diversity from different regions was similar only in the second period but significantly different in the third and fourth periods. The diversity of fungi and bacteria was different, which was influenced by the region. This result was consistent with a study on fresh hairtails from different regions that reported that *Psychrobacter*, *Thermus* and *Oceanisphaera* were the dominant bacteria in samples from Nantong, while a wide variety of bacterial genera were found in samples from Zhoushan (Qiankun et al., 2018). Another study of water areas produced similar results, with samples from the western part of Shenzhen containing more *Proclococcus* and samples from the eastern part of Shenzhen containing more *Chlorella* (Zhang et al., 2021). This results showed that there were differences in microorganisms of diseased potato tubers in different ecological regions which may be related to the local natural environment.

The co-occurrence network analysis of plant microbial communities could provide a new perspective for strengthening disease management and identifying candidate microorganisms afecting plant health (Poudel et al., 2016). The results showed that there were three cases interaction between pathogens and antagonists in diseased potato tubers. In most cases, no matter between fungi or bacteria, there was a negative correlation between pathogens and antagonistic microbe, and a positive correlation between antagonistic bacteria, while there was both a positive correlation and a negative correlation between pathogenic fungi. Moreover, it was worth noting that in the interaction between fungi and bacteria, there was a negative correlation between pathogenic fungi *Fusarium* and all screened bacteria while the pathogenic bacteria *Erwinia* was negatively correlated with all the selected fungi, this may indicate that the pathogenic fungi and pathogenic bacteria in diseased potatoes show a pattern of one growth and the other decline, which is also consistent with the disease development of fungal dry rot in the early stage of storage and bacterial soft rot in the later stage of storage. As *Pseudomonas*, which we mentioned in the previous discussion may be both pathogenic bacteria and antimicrobial bacteria, we found that it was negatively correlated with pathogen *Erwinia* and positively correlated with antagonistic bacteria *Bacillus* (Figure 10B), and it also has a strong negative correlation with *Fusarium* and a positive correlation with antagonistic fungi (Figure 10C), so it was speculated that *Pseudomonas* here is antagonistic bacteria. A similar situation occurs in the interaction of *Alternaria* that there was a positive correlation between *Alternaria* and *Bacillus* (Figure 10C), and *Erwinia* had a strong negative correlation with *Mucor* and *Alternaria*, while *Mucor* positively correlated with *Alternaria* (Figure 10A), indicating that *Alternaria* may be an antagonistic fungi. It is reported that *Alternaria* is an endophyte of many plants (Kaur et al., 2020), as the pathogen of potato early blight, it mainly infects leaves, and in this study, *Alternaria* mainly exists in the samples of the first stage of the cellar period, so it may exist mainly in the form of endophytes, or in the form of pathogens in the early stage of cellar, so it shows a complex relationship. Trough the microbial interaction network, we can identify and obtain microorganisms that are antagonistic to pathogens (Poudel et al., 2016). Therefore, the correlation analysis of co-occurrence network in our study can be used as a basis for preliminary judgment of the interaction between pathogens and antagonists, but for the genera that belong to both antagonistic bacteria and pathogens, it is also necessary to combine the distribution of species and related studies to judge and verify.

Tubers are initially or externally infected with spores in the field. Even a small quantity of diseased tubers in a lot can potentially result in the disease spreading and the whole lot being damaged during storage. Harvesting, handling, and transportation may also lead to mechanical damage to tubers, which can result in the entry of pathogenic organisms. It is recommended that tubers be examined for pests and diseases prior to storage. Grading of tubers during postharvest storage of potatoes is important before storage to eliminate diseased or infected tubers (Pinhero et al., 2009). In our experiment, healthy postharvest potatoes were selected and dried for 7 days for pre-storage, while those that were not picked and dried were stored directly as controls. In terms of whether the treatment could improve the storage quality of food, some studies have proven that spices and packaging film can delay the lag period of bacterial growth and extend the shelf life of pot-stewed duck wings during storage by using a high-throughput sequencing method (Ye et al., 2017). In our study, the selection and drying treatment reduced the richness and diversity of microorganisms in diseased potato tubers, especially the relative abundance of pathogens of potato and other plants, proving that pre-storage drying could be an effective treatment for potato cellaring.

## Conclusion

The microbial community structure and diversity of diseased potato tubers were analyzed by high-throughput sequencing. The results showed that the community structure and diversity of microbes in different regions and different cellaring periods were varies. The dominant fungal phylum was *Proteobacteria* of *Ascomycota* in HY, MY and ZL and *Apiotrichum* of *Basidiomycota* in HZ. The dominant bacterial phyla were all *Pseudomonas* of *Proteobacteria*, but their abundance varied in samples from different regions and varied with storage time. In the analysis of indicators, there were some common species and endemic species in different regions and periods, and the abundance of some fungi, especially *Fusarium* and other potato pathogens, was higher in Group “C” than in Group “A.” In the diversity analysis, the abundance of fungal α diversity in Group “C” was higher than that in Group “A.” However, the abundance of bacterial α diversity in “A” was higher than that in “C,” and there was no obvious regularity with aging time. In addition, through functional prediction analysis, it was found that the main nutritional types of fungi were Pathotroph-Saprotroph-Symbiotroph, Pathotroph-Symbiotroph and Saprotroph, which indicated that treatment consisting of selection and drying could significantly reduce the plant pathogenic microbes and other microorganisms of diseased potato tubers, and it could be an effective measure for potato storage. More importantly, the microbial interaction network showed a negative correlation between pathogens and antagonistic bacteria that can identify and obtain microorganisms that are antagonistic to pathogens. The pathogenicity of pathogenic microbes and the bacteriostatic effect of antagonistic microbes on potato will be further studied. This study provided a theoretical basis for maintaining potato quality during long-term cellaring.

## Data Availability

The datasets presented in this study can be found in online repositories. The names of the repository/repositories and accession number(s) can be found below: https://www.ncbi.nlm.nih.gov/, SRR16771893–SRR16772017 https://www.ncbi.nlm.nih.gov/, SRR16643766–SRR16643890.
