# Supplementary material for: Comparative Analysis of Microbial Community Diversity and Dynamics on Diseased Tubers During Potato Storage in Different Regions of Qinghai China
Source: Front Genet. 2022 Feb 22;13:818940. doi: 10.3389/fgene.2022.818940 (PMC8902257; doi:10.3389/fgene.2022.818940)
Supplement: Supplementary file 2 [file DataSheet2.docx]

**Supplementary Table 1.** The sequencing data of fungi on the samples of diseased potato tubers

| **Samples** | **Raw PE** | **Clean PE** | **Samples** | **Raw PE** | **Clean PE** |
| --- | --- | --- | --- | --- | --- |
| HY-1-C-1 | 129128 | 128776 | ZL-1-C-1 | 124956 | 124696 |
| HY-1-C-2 | 126014 | 125626 | ZL-1-C-2 | 121992 | 121764 |
| HY-1-C-3 | 125283 | 124925 | ZL-1-C-3 | 136079 | 136014 |
| HY-1-C-4 | 120631 | 120251 | ZL-1-C-4 | 126495 | 126430 |
| HY-1-C-5 | 121857 | 121416 | ZL-1-C-5 | 120421 | 120369 |
| HY-2-A-1 | 128529 | 128505 | ZL-2-A-1 | 126529 | 126501 |
| HY-2-A-2 | 133444 | 133415 | ZL-2-A-2 | 135203 | 135168 |
| HY-2-A-3 | 128263 | 128244 | ZL-2-A-3 | 128618 | 128595 |
| HY-2-A-4 | 121366 | 121337 | ZL-2-A-4 | 121393 | 121362 |
| HY-2-A-5 | 131911 | 131892 | ZL-2-A-5 | 126896 | 126877 |
| HY-2-C-1 | 130294 | 129979 | ZL-2-C-1 | 136575 | 136545 |
| HY-2-C-2 | 131904 | 131570 | ZL-2-C-2 | 137082 | 137046 |
| HY-2-C-3 | 133808 | 133435 | ZL-2-C-3 | 122507 | 122492 |
| HY-2-C-4 | 133512 | 133095 | ZL-2-C-4 | 125312 | 125282 |
| HY-2-C-5 | 120536 | 120130 | ZL-2-C-5 | 136115 | 136078 |
| HY-3-A-1 | 133333 | 133289 | ZL-3-A-1 | 135884 | 135847 |
| HY-3-A-2 | 133510 | 133455 | ZL-3-A-2 | 133658 | 133600 |
| HY-3-A-3 | 127875 | 127842 | ZL-3-A-3 | 129865 | 129820 |
| HY-3-A-4 | 128775 | 128727 | ZL-3-A-4 | 131331 | 131296 |
| HY-3-A-5 | 136232 | 136180 | ZL-3-A-5 | 132138 | 132087 |
| HY-3-C-1 | 130226 | 130167 | ZL-3-C-1 | 127760 | 127713 |
| HY-3-C-2 | 127223 | 127172 | ZL-3-C-2 | 126451 | 126409 |
| HY-3-C-3 | 129784 | 129732 | ZL-3-C-3 | 132278 | 132219 |
| HY-3-C-4 | 129905 | 129861 | ZL-3-C-4 | 122052 | 122000 |
| HY-3-C-5 | 124806 | 124767 | ZL-3-C-5 | 137025 | 136967 |
| HY-4-A-1 | 130459 | 130242 | ZL-4-A-1 | 129816 | 129631 |
| HY-4-A-2 | 130565 | 130370 | ZL-4-A-2 | 123512 | 123333 |
| HY-4-A-3 | 137414 | 137215 | ZL-4-A-3 | 124131 | 123968 |
| HY-4-A-4 | 125046 | 124857 | ZL-4-A-4 | 127046 | 126882 |
| HY-4-A-5 | 135310 | 135080 | ZL-4-A-5 | 120313 | 120071 |
| HY-4-C-1 | 130521 | 130281 | ZL-4-C-1 | 132505 | 132323 |
| HY-4-C-2 | 135285 | 135065 | ZL-4-C-2 | 127092 | 126947 |
| HY-4-C-3 | 128608 | 128349 | ZL-4-C-3 | 129158 | 128955 |
| HY-4-C-4 | 136142 | 135897 | ZL-4-C-4 | 129018 | 128799 |
| HY-4-C-5 | 121060 | 120862 | ZL-4-C-5 | 131680 | 131458 |
| MY-1-C-1 | 137087 | 136819 | HZ-1-C-1 | 134021 | 133736 |
| MY-1-C-2 | 132396 | 132133 | HZ-1-C-2 | 136842 | 136552 |
| MY-1-C-3 | 120421 | 120217 | HZ-1-C-3 | 124666 | 124347 |
| MY-1-C-4 | 128653 | 128429 | HZ-1-C-4 | 126742 | 126394 |
| MY-1-C-5 | 124726 | 124483 | HZ-1-C-5 | 122581 | 122275 |
| MY-2-A-1 | 134499 | 134471 | HZ-2-C-1 | 133058 | 133040 |
| MY-2-A-2 | 122325 | 122303 | HZ-2-C-2 | 124487 | 124464 |
| MY-2-A-3 | 124634 | 124610 | HZ-2-C-3 | 134763 | 134731 |
| MY-2-A-4 | 128501 | 128482 | HZ-2-C-4 | 124523 | 124507 |
| MY-2-A-5 | 124057 | 124035 | HZ-2-C-5 | 127838 | 127805 |
| MY-2-C-1 | 137506 | 137481 | HZ-3-C-1 | 124482 | 124446 |
| MY-2-C-2 | 123199 | 123169 | HZ-3-C-2 | 119722 | 119681 |
| MY-2-C-3 | 120792 | 120773 | HZ-3-C-3 | 136706 | 136643 |
| MY-2-C-4 | 122601 | 122577 | HZ-3-C-4 | 135649 | 135596 |
| MY-2-C-5 | 136643 | 136616 | HZ-3-C-5 | 124266 | 124207 |
| MY-3-A-1 | 123579 | 123521 | HZ-4-C-1 | 121318 | 121156 |
| MY-3-A-2 | 121574 | 121523 | HZ-4-C-2 | 129888 | 129708 |
| MY-3-A-3 | 130875 | 130813 | HZ-4-C-3 | 129451 | 129258 |
| MY-3-A-4 | 130523 | 130477 | HZ-4-C-4 | 136693 | 136541 |
| MY-3-A-5 | 127202 | 127153 | HZ-4-C-5 | 136382 | 136250 |
| MY-3-C-1 | 136656 | 136608 |  |  |  |
| MY-3-C-2 | 121049 | 120966 |  |  |  |
| MY-3-C-3 | 131050 | 130968 |  |  |  |
| MY-3-C-4 | 134126 | 134045 |  |  |  |
| MY-3-C-5 | 128619 | 128549 |  |  |  |
| MY-4-A-1 | 123491 | 123316 |  |  |  |
| MY-4-A-2 | 124056 | 123893 |  |  |  |
| MY-4-A-3 | 122872 | 122696 |  |  |  |
| MY-4-A-4 | 131982 | 131787 |  |  |  |
| MY-4-A-5 | 135265 | 135068 |  |  |  |
| MY-4-C-1 | 124102 | 123838 |  |  |  |
| MY-4-C-2 | 130631 | 130401 |  |  |  |
| MY-4-C-3 | 121054 | 120841 |  |  |  |
| MY-4-C-4 | 123375 | 123148 |  |  |  |
| MY-4-C-5 | 122601 | 122403 |  |  |  |

**Supplementary Table 2.** The sequencing data of bacteria on the samples of diseased potato tubers

| **Samples** | **Raw PE** | **Clean PE** | **Samples** | **Raw PE** | **Clean PE** |
| --- | --- | --- | --- | --- | --- |
| HY-1-C-1 | 131613 | 131197 | ZL-1-C-1 | 134230 | 133929 |
| HY-1-C-2 | 125181 | 124784 | ZL-1-C-2 | 132099 | 131614 |
| HY-1-C-3 | 126553 | 126032 | ZL-1-C-3 | 125496 | 125048 |
| HY-1-C-4 | 131181 | 130693 | ZL-1-C-4 | 137235 | 136752 |
| HY-1-C-5 | 122720 | 122265 | ZL-1-C-5 | 135392 | 134997 |
| HY-2-A-1 | 101236 | 101154 | ZL-2-A-1 | 122651 | 122391 |
| HY-2-A-2 | 137698 | 137028 | ZL-2-A-2 | 122131 | 122071 |
| HY-2-A-3 | 121145 | 120175 | ZL-2-A-3 | 126119 | 126063 |
| HY-2-A-4 | 125020 | 124007 | ZL-2-A-4 | 129973 | 129918 |
| HY-2-A-5 | 125697 | 124707 | ZL-2-A-5 | 128268 | 128216 |
| HY-2-C-1 | 124194 | 123822 | ZL-2-C-1 | 123144 | 123088 |
| HY-2-C-2 | 135577 | 135199 | ZL-2-C-2 | 133305 | 133254 |
| HY-2-C-3 | 126606 | 126261 | ZL-2-C-3 | 132911 | 132853 |
| HY-2-C-4 | 129630 | 129317 | ZL-2-C-4 | 120800 | 120753 |
| HY-2-C-5 | 123372 | 123060 | ZL-2-C-5 | 135847 | 135758 |
| HY-3-A-1 | 127075 | 126998 | ZL-3-A-1 | 131947 | 131845 |
| HY-3-A-2 | 123276 | 123204 | ZL-3-A-2 | 120526 | 120464 |
| HY-3-A-3 | 129768 | 129695 | ZL-3-A-3 | 135608 | 135536 |
| HY-3-A-4 | 119597 | 119535 | ZL-3-A-4 | 130320 | 130247 |
| HY-3-A-5 | 130435 | 130353 | ZL-3-A-5 | 137843 | 137767 |
| HY-3-C-1 | 123493 | 123433 | ZL-3-C-1 | 130115 | 130058 |
| HY-3-C-2 | 133296 | 133222 | ZL-3-C-2 | 127642 | 127571 |
| HY-3-C-3 | 127197 | 127127 | ZL-3-C-3 | 124534 | 124477 |
| HY-3-C-4 | 114040 | 113967 | ZL-3-C-4 | 124197 | 124116 |
| HY-3-C-5 | 115238 | 115176 | ZL-3-C-5 | 131660 | 131579 |
| HY-4-A-1 | 123136 | 122789 | ZL-4-A-1 | 134586 | 134246 |
| HY-4-A-2 | 137471 | 137124 | ZL-4-A-2 | 128750 | 128410 |
| HY-4-A-3 | 128547 | 128246 | ZL-4-A-3 | 130855 | 130536 |
| HY-4-A-4 | 126521 | 126183 | ZL-4-A-4 | 123299 | 122979 |
| HY-4-A-5 | 121944 | 121647 | ZL-4-A-5 | 123876 | 123589 |
| HY-4-C-1 | 130931 | 130645 | ZL-4-C-1 | 132728 | 132443 |
| HY-4-C-2 | 128292 | 127994 | ZL-4-C-2 | 124182 | 123870 |
| HY-4-C-3 | 121100 | 120785 | ZL-4-C-3 | 125838 | 125573 |
| HY-4-C-4 | 125845 | 125541 | ZL-4-C-4 | 129397 | 129090 |
| HY-4-C-5 | 129382 | 129115 | ZL-4-C-5 | 137720 | 137424 |
| MY-1-C-1 | 129065 | 128494 | HZ-1-C-1 | 122082 | 121799 |
| MY-1-C-2 | 124615 | 123808 | HZ-1-C-2 | 135729 | 135330 |
| MY-1-C-3 | 135552 | 134869 | HZ-1-C-3 | 122043 | 121588 |
| MY-1-C-4 | 123909 | 123165 | HZ-1-C-4 | 128564 | 127929 |
| MY-1-C-5 | 121563 | 121221 | HZ-1-C-5 | 132254 | 131978 |
| MY-2-A-1 | 130990 | 130929 | HZ-2-C-1 | 133194 | 133084 |
| MY-2-A-2 | 131283 | 130433 | HZ-2-C-2 | 124806 | 124747 |
| MY-2-A-3 | 135479 | 134504 | HZ-2-C-3 | 126499 | 126436 |
| MY-2-A-4 | 132693 | 129689 | HZ-2-C-4 | 131604 | 131477 |
| MY-2-A-5 | 72930 | 72399 | HZ-2-C-5 | 131436 | 131386 |
| MY-2-C-1 | 133248 | 133198 | HZ-3-C-1 | 126731 | 126639 |
| MY-2-C-2 | 124890 | 124780 | HZ-3-C-2 | 121421 | 121338 |
| MY-2-C-3 | 132937 | 132729 | HZ-3-C-3 | 125326 | 125239 |
| MY-2-C-4 | 136214 | 135973 | HZ-3-C-4 | 134664 | 134553 |
| MY-2-C-5 | 129036 | 128935 | HZ-3-C-5 | 126588 | 126522 |
| MY-3-A-1 | 120925 | 120857 | HZ-4-C-1 | 121941 | 121622 |
| MY-3-A-2 | 133584 | 133314 | HZ-4-C-2 | 128607 | 128249 |
| MY-3-A-3 | 123047 | 122679 | HZ-4-C-3 | 121013 | 120690 |
| MY-3-A-4 | 124514 | 124353 | HZ-4-C-4 | 131613 | 131280 |
| MY-3-A-5 | 136434 | 136344 | HZ-4-C-5 | 134741 | 134466 |
| MY-3-C-1 | 123881 | 123825 |  |  |  |
| MY-3-C-2 | 120594 | 120498 |  |  |  |
| MY-3-C-3 | 103109 | 103041 |  |  |  |
| MY-3-C-4 | 131373 | 131194 |  |  |  |
| MY-3-C-5 | 128104 | 128041 |  |  |  |
| MY-4-A-1 | 123113 | 122831 |  |  |  |
| MY-4-A-2 | 123803 | 123212 |  |  |  |
| MY-4-A-3 | 121708 | 121148 |  |  |  |
| MY-4-A-4 | 122367 | 121958 |  |  |  |
| MY-4-A-5 | 136216 | 135897 |  |  |  |
| MY-4-C-1 | 127176 | 126874 |  |  |  |
| MY-4-C-2 | 130846 | 130296 |  |  |  |
| MY-4-C-3 | 137022 | 136408 |  |  |  |
| MY-4-C-4 | 123462 | 123114 |  |  |  |
| MY-4-C-5 | 130743 | 130435 |  |  |  |

**Supplementary Table 3.** The OUT numbers of fungi on the samples of diseased potato tubers

| **Samples in HY** | **OTUs** | **Samples in MY** | **OTUs** | **Samples in ZL** | **OTUs** | **Samples in HZ** | **OTUs** |
| --- | --- | --- | --- | --- | --- | --- | --- |
| HY-1-C-1 | 210 | MY-1-C-1 | 164 | ZL-1-C-1 | 155 | HZ-1-C-1 | 166 |
| HY-1-C-2 | 217 | MY-1-C-2 | 156 | ZL-1-C-2 | 131 | HZ-1-C-2 | 125 |
| HY-1-C-3 | 154 | MY-1-C-3 | 97 | ZL-1-C-3 | 122 | HZ-1-C-3 | 138 |
| HY-1-C-4 | 173 | MY-1-C-4 | 139 | ZL-1-C-4 | 111 | HZ-1-C-4 | 144 |
| HY-1-C-5 | 239 | MY-1-C-5 | 141 | ZL-1-C-5 | 113 | HZ-1-C-5 | 170 |
| HY-2-A-1 | 76 | MY-2-A-1 | 158 | ZL-2-A-1 | 157 | HZ-2-C-1 | 99 |
| HY-2-A-2 | 87 | MY-2-A-2 | 125 | ZL-2-A-2 | 174 | HZ-2-C-2 | 140 |
| HY-2-A-3 | 77 | MY-2-A-3 | 154 | ZL-2-A-3 | 161 | HZ-2-C-3 | 147 |
| HY-2-A-4 | 90 | MY-2-A-4 | 104 | ZL-2-A-4 | 114 | HZ-2-C-4 | 136 |
| HY-2-A-5 | 77 | MY-2-A-5 | 158 | ZL-2-A-5 | 133 | HZ-2-C-5 | 141 |
| HY-2-C-1 | 182 | MY-2-C-1 | 111 | ZL-2-C-1 | 164 | HZ-3-C-1 | 143 |
| HY-2-C-2 | 140 | MY-2-C-2 | 158 | ZL-2-C-2 | 240 | HZ-3-C-2 | 118 |
| HY-2-C-3 | 157 | MY-2-C-3 | 80 | ZL-2-C-3 | 127 | HZ-3-C-3 | 94 |
| HY-2-C-4 | 105 | MY-2-C-4 | 110 | ZL-2-C-4 | 158 | HZ-3-C-4 | 124 |
| HY-2-C-5 | 116 | MY-2-C-5 | 156 | ZL-2-C-5 | 170 | HZ-3-C-5 | 78 |
| HY-3-A-1 | 103 | MY-3-A-1 | 103 | ZL-3-A-1 | 112 | HZ-4-C-1 | 121 |
| HY-3-A-2 | 176 | MY-3-A-2 | 113 | ZL-3-A-2 | 99 | HZ-4-C-2 | 151 |
| HY-3-A-3 | 96 | MY-3-A-3 | 119 | ZL-3-A-3 | 98 | HZ-4-C-3 | 117 |
| HY-3-A-4 | 89 | MY-3-A-4 | 194 | ZL-3-A-4 | 112 | HZ-4-C-4 | 84 |
| HY-3-A-5 | 116 | MY-3-A-5 | 143 | ZL-3-A-5 | 122 | HZ-4-C-5 | 115 |
| HY-3-C-1 | 137 | MY-3-C-1 | 188 | ZL-3-C-1 | 137 |  |  |
| HY-3-C-2 | 176 | MY-3-C-2 | 201 | ZL-3-C-2 | 106 |  |  |
| HY-3-C-3 | 182 | MY-3-C-3 | 161 | ZL-3-C-3 | 121 |  |  |
| HY-3-C-4 | 138 | MY-3-C-4 | 152 | ZL-3-C-4 | 136 |  |  |
| HY-3-C-5 | 112 | MY-3-C-5 | 125 | ZL-3-C-5 | 130 |  |  |
| HY-4-A-1 | 86 | MY-4-A-1 | 102 | ZL-4-A-1 | 99 |  |  |
| HY-4-A-2 | 88 | MY-4-A-2 | 93 | ZL-4-A-2 | 116 |  |  |
| HY-4-A-3 | 102 | MY-4-A-3 | 114 | ZL-4-A-3 | 74 |  |  |
| HY-4-A-4 | 86 | MY-4-A-4 | 76 | ZL-4-A-4 | 90 |  |  |
| HY-4-A-5 | 97 | MY-4-A-5 | 105 | ZL-4-A-5 | 88 |  |  |
| HY-4-C-1 | 74 | MY-4-C-1 | 90 | ZL-4-C-1 | 161 |  |  |
| HY-4-C-2 | 97 | MY-4-C-2 | 138 | ZL-4-C-2 | 107 |  |  |
| HY-4-C-3 | 83 | MY-4-C-3 | 108 | ZL-4-C-3 | 111 |  |  |
| HY-4-C-4 | 97 | MY-4-C-4 | 90 | ZL-4-C-4 | 129 |  |  |
| HY-4-C-5 | 114 | MY-4-C-5 | 117 | ZL-4-C-5 | 147 |  |  |

**Supplementary Table 4.** The OUT numbers of bacteria on the samples of diseased potato tubers

| **Samples in HY** | **OTUs** | **Samples in MY** | **OTUs** | **Samples in ZL** | **OTUs** | **HZ Samples** | **OTUs** |
| --- | --- | --- | --- | --- | --- | --- | --- |
| HY-1-C-1 | 463 | MY-1-C-1 | 446 | ZL-1-C-1 | 368 | HZ-1-C-1 | 288 |
| HY-1-C-2 | 559 | MY-1-C-2 | 193 | ZL-1-C-2 | 323 | HZ-1-C-2 | 297 |
| HY-1-C-3 | 395 | MY-1-C-3 | 200 | ZL-1-C-3 | 221 | HZ-1-C-3 | 296 |
| HY-1-C-4 | 438 | MY-1-C-4 | 151 | ZL-1-C-4 | 233 | HZ-1-C-4 | 240 |
| HY-1-C-5 | 837 | MY-1-C-5 | 215 | ZL-1-C-5 | 143 | HZ-1-C-5 | 452 |
| HY-2-A-1 | 459 | MY-2-A-1 | 629 | ZL-2-A-1 | 554 | HZ-2-C-1 | 420 |
| HY-2-A-2 | 440 | MY-2-A-2 | 659 | ZL-2-A-2 | 618 | HZ-2-C-2 | 443 |
| HY-2-A-3 | 301 | MY-2-A-3 | 434 | ZL-2-A-3 | 347 | HZ-2-C-3 | 470 |
| HY-2-A-4 | 452 | MY-2-A-4 | 717 | ZL-2-A-4 | 370 | HZ-2-C-4 | 456 |
| HY-2-A-5 | 664 | MY-2-A-5 | 534 | ZL-2-A-5 | 324 | HZ-2-C-5 | 301 |
| HY-2-C-1 | 395 | MY-2-C-1 | 377 | ZL-2-C-1 | 628 | HZ-3-C-1 | 534 |
| HY-2-C-2 | 376 | MY-2-C-2 | 231 | ZL-2-C-2 | 334 | HZ-3-C-2 | 402 |
| HY-2-C-3 | 264 | MY-2-C-3 | 270 | ZL-2-C-3 | 313 | HZ-3-C-3 | 466 |
| HY-2-C-4 | 355 | MY-2-C-4 | 462 | ZL-2-C-4 | 274 | HZ-3-C-4 | 451 |
| HY-2-C-5 | 356 | MY-2-C-5 | 423 | ZL-2-C-5 | 380 | HZ-3-C-5 | 336 |
| HY-3-A-1 | 554 | MY-3-A-1 | 682 | ZL-3-A-1 | 465 | HZ-4-C-1 | 479 |
| HY-3-A-2 | 419 | MY-3-A-2 | 736 | ZL-3-A-2 | 473 | HZ-4-C-2 | 465 |
| HY-3-A-3 | 409 | MY-3-A-3 | 658 | ZL-3-A-3 | 456 | HZ-4-C-3 | 432 |
| HY-3-A-4 | 688 | MY-3-A-4 | 606 | ZL-3-A-4 | 318 | HZ-4-C-4 | 378 |
| HY-3-A-5 | 575 | MY-3-A-5 | 324 | ZL-3-A-5 | 313 | HZ-4-C-5 | 346 |
| HY-3-C-1 | 509 | MY-3-C-1 | 462 | ZL-3-C-1 | 420 |  |  |
| HY-3-C-2 | 569 | MY-3-C-2 | 526 | ZL-3-C-2 | 496 |  |  |
| HY-3-C-3 | 432 | MY-3-C-3 | 312 | ZL-3-C-3 | 332 |  |  |
| HY-3-C-4 | 461 | MY-3-C-4 | 358 | ZL-3-C-4 | 275 |  |  |
| HY-3-C-5 | 561 | MY-3-C-5 | 319 | ZL-3-C-5 | 309 |  |  |
| HY-4-A-1 | 533 | MY-4-A-1 | 411 | ZL-4-A-1 | 459 |  |  |
| HY-4-A-2 | 477 | MY-4-A-2 | 460 | ZL-4-A-2 | 452 |  |  |
| HY-4-A-3 | 562 | MY-4-A-3 | 462 | ZL-4-A-3 | 514 |  |  |
| HY-4-A-4 | 391 | MY-4-A-4 | 393 | ZL-4-A-4 | 430 |  |  |
| HY-4-A-5 | 360 | MY-4-A-5 | 488 | ZL-4-A-5 | 313 |  |  |
| HY-4-C-1 | 352 | MY-4-C-1 | 386 | ZL-4-C-1 | 720 |  |  |
| HY-4-C-2 | 486 | MY-4-C-2 | 573 | ZL-4-C-2 | 421 |  |  |
| HY-4-C-3 | 342 | MY-4-C-3 | 445 | ZL-4-C-3 | 651 |  |  |
| HY-4-C-4 | 379 | MY-4-C-4 | 372 | ZL-4-C-4 | 368 |  |  |
| HY-4-C-5 | 370 | MY-4-C-5 | 365 | ZL-4-C-5 | 432 |  |  |

**Supplementary Table 5.** The alpha diversity index of fungal community the samples of diseased potato tubers

| **group** | **sobs** | **shannon** | **simpson** | **chao** | **ace** | **goods_coverage** | **pielou** | **pd** |
| --- | --- | --- | --- | --- | --- | --- | --- | --- |
| HY-1-C | 198.6 | 2.8819 | 0.693311 | 235.6276 | 234.6557 | 0.9996506 | 0.379345 | 54.0515 |
| HY-2-A | 81.4 | 1.150885 | 0.33071 | 103.3448 | 110.2965 | 0.9998153 | 0.180582 | 20.6691 |
| HY-2-C | 140 | 1.720511 | 0.491812 | 192.7193 | 189.7716 | 0.9996549 | 0.238993 | 41.53152 |
| HY-3-A | 116 | 1.647336 | 0.467972 | 149.1924 | 153.1253 | 0.9997593 | 0.238995 | 29.32241 |
| HY-3-C | 149 | 1.816412 | 0.439495 | 183.5794 | 187.5738 | 0.9997127 | 0.247508 | 35.36171 |
| HY-4-A | 91.8 | 1.873041 | 0.538633 | 115.2583 | 115.0392 | 0.9998196 | 0.287345 | 25.34224 |
| HY-4-C | 93 | 2.170486 | 0.647374 | 118.6389 | 121.1289 | 0.9997983 | 0.33302 | 25.57348 |
| HZ-1-C | 148.6 | 3.055628 | 0.743985 | 180.8256 | 187.4149 | 0.9997159 | 0.424455 | 47.10363 |
| HZ-2-C | 132.6 | 1.989398 | 0.561781 | 186.8084 | 181.2162 | 0.9996744 | 0.280144 | 34.64351 |
| HZ-3-C | 111.4 | 1.516456 | 0.409717 | 137.9837 | 137.8432 | 0.9997923 | 0.219399 | 29.28559 |
| HZ-4-C | 117.6 | 2.157873 | 0.563998 | 138.68 | 136.271 | 0.9998124 | 0.313023 | 32.49986 |
| MY-1-C | 139.4 | 2.625551 | 0.660686 | 177.0336 | 169.1541 | 0.999733 | 0.367227 | 40.85405 |
| MY-2-A | 139.8 | 2.116997 | 0.537286 | 171.7888 | 170.4783 | 0.9997634 | 0.295287 | 31.76087 |
| MY-2-C | 123 | 2.268343 | 0.636389 | 147.327 | 151.3303 | 0.9997866 | 0.326201 | 29.59187 |
| MY-3-A | 134.4 | 1.785848 | 0.473163 | 169.9544 | 168.084 | 0.9997287 | 0.248168 | 33.28396 |
| MY-3-C | 165.4 | 3.047886 | 0.76659 | 198.8758 | 196.6456 | 0.9997381 | 0.413729 | 39.4527 |
| MY-4-A | 98 | 1.242021 | 0.295646 | 128.9316 | 127.741 | 0.9997882 | 0.187728 | 27.90363 |
| MY-4-C | 108.6 | 2.421739 | 0.703718 | 139.3825 | 135.6964 | 0.9997749 | 0.358091 | 30.14906 |
| ZL-1-C | 126.4 | 2.244876 | 0.559055 | 146.7158 | 153.2023 | 0.9997846 | 0.319123 | 43.55124 |
| ZL-2-A | 147.8 | 2.689039 | 0.690178 | 188.5662 | 192.5341 | 0.9996854 | 0.371766 | 35.76434 |
| ZL-2-C | 171.8 | 3.097514 | 0.719338 | 210.1282 | 214.5162 | 0.9996926 | 0.414574 | 38.97742 |
| ZL-3-A | 108.6 | 1.638545 | 0.437102 | 136.5078 | 133.9293 | 0.9998007 | 0.242958 | 28.49424 |
| ZL-3-C | 126 | 2.39873 | 0.596171 | 154.6151 | 156.3248 | 0.999784 | 0.342937 | 30.48417 |
| ZL-4-A | 93.4 | 1.805338 | 0.4928 | 106.7112 | 110.9048 | 0.9998383 | 0.274513 | 23.90784 |
| ZL-4-C | 131 | 2.296663 | 0.572513 | 151.8367 | 158.1259 | 0.9997717 | 0.323945 | 33.19571 |

**Supplementary Table 6.** The alpha diversity index of bacteria community the samples of diseased potato tubers

| **group** | **sobs** | **shannon** | **simpson** | **chao** | **ace** | **Goods coverage** | **pielou** | **pd** |
| --- | --- | --- | --- | --- | --- | --- | --- | --- |
| HY-1-C | 538.4 | 4.156262 | 0.875026 | 759.6807 | 781.064 | 0.99769 | 0.459354 | 82.96583 |
| HY-2-A | 463.2 | 2.920964 | 0.64564 | 595.77 | 600.4689 | 0.998555 | 0.330821 | 97.24763 |
| HY-2-C | 349.2 | 3.429148 | 0.821803 | 522.7614 | 529.6477 | 0.998552 | 0.406351 | 74.1622 |
| HY-3-A | 529 | 3.665324 | 0.804662 | 716.0128 | 716.5038 | 0.998138 | 0.405287 | 94.10886 |
| HY-3-C | 506.4 | 3.25687 | 0.750497 | 680.3294 | 698.1306 | 0.998032 | 0.362159 | 81.99293 |
| HY-4-A | 464.6 | 3.351576 | 0.762945 | 624.8611 | 626.8441 | 0.99816 | 0.378898 | 86.46726 |
| HY-4-C | 385.8 | 2.964411 | 0.742886 | 539.8859 | 541.6974 | 0.998401 | 0.345054 | 77.43494 |
| HZ-1-C | 314.6 | 2.708191 | 0.676014 | 413.5104 | 428.5652 | 0.999046 | 0.327034 | 60.50181 |
| HZ-2-C | 418 | 4.26937 | 0.873553 | 543.1886 | 561.0117 | 0.998162 | 0.489416 | 76.32318 |
| HZ-3-C | 437.8 | 4.433504 | 0.891481 | 556.6151 | 561.7397 | 0.998508 | 0.50494 | 75.23936 |
| HZ-4-C | 420 | 3.89797 | 0.843996 | 520.8088 | 543.0472 | 0.998332 | 0.447278 | 69.33838 |
| MY-1-C | 241 | 2.762579 | 0.686858 | 340.6445 | 324.2252 | 0.999273 | 0.35575 | 46.34231 |
| MY-2-A | 594.6 | 3.581766 | 0.741544 | 764.7267 | 779.1925 | 0.997725 | 0.389935 | 105.6298 |
| MY-2-C | 352.6 | 2.430059 | 0.651911 | 472.2791 | 478.3661 | 0.998897 | 0.287621 | 71.11685 |
| MY-3-A | 601.2 | 3.920977 | 0.829019 | 778.6778 | 814.1472 | 0.997752 | 0.426225 | 98.45463 |
| MY-3-C | 395.4 | 3.174758 | 0.743542 | 525.6518 | 554.5709 | 0.998541 | 0.367711 | 76.8328 |
| MY-4-A | 442.8 | 3.849801 | 0.815598 | 600.4541 | 620.6485 | 0.997957 | 0.43791 | 85.86516 |
| MY-4-C | 428.2 | 3.532643 | 0.754746 | 559.4677 | 572.971 | 0.998278 | 0.403129 | 69.09994 |
| ZL-1-C | 257.6 | 2.486613 | 0.637738 | 337.8639 | 330.2164 | 0.999303 | 0.309782 | 52.89872 |
| ZL-2-A | 442.6 | 2.810013 | 0.665894 | 610.5575 | 629.9008 | 0.998293 | 0.320662 | 84.15235 |
| ZL-2-C | 385.8 | 2.493985 | 0.65198 | 554.7852 | 572.1511 | 0.998364 | 0.292475 | 81.37501 |
| ZL-3-A | 405 | 2.341196 | 0.552621 | 529.9501 | 538.9754 | 0.998757 | 0.270644 | 80.43161 |
| ZL-3-C | 366.4 | 3.129181 | 0.765915 | 519.976 | 519.9687 | 0.998659 | 0.367042 | 78.23652 |
| ZL-4-A | 433.6 | 3.680614 | 0.818883 | 562.1513 | 575.1359 | 0.998282 | 0.420845 | 76.06843 |
| ZL-4-C | 518.4 | 3.425121 | 0.773766 | 665.7915 | 683.108 | 0.997964 | 0.380502 | 92.94444 |

**Supplementary Table 7.** The main nutritional types of fungi of diseased potato at the level of guild classification

| **Guilds** | **Total_tags** |
| --- | --- |
| Plant Pathogen | 989293 |
| Animal Pathogen | 17140 |
| Animal Parasite-Fungal Parasite | 449 |
| Fungal Parasite | 17 |
| Animal Pathogen-Plant Pathogen-Undefined Saprotroph | 248409 |
| Dung Saprotroph-Plant Pathogen | 13702 |
| Animal Endosymbiont-Animal Pathogen-Endophyte-Plant Pathogen-Undefined Saprotroph | 5266 |
| Plant Pathogen-Undefined Saprotroph | 2283 |
| Fungal Parasite-Plant Pathogen-Plant Saprotroph | 1587 |
| Algal Parasite-Bryophyte Parasite-Fungal Parasite-Undefined Saprotroph | 1060 |
| Animal Pathogen-Undefined Saprotroph | 809 |
| Plant Pathogen-Wood Saprotroph | 415 |
| Fungal Parasite-Undefined Saprotroph | 315 |
| Animal Pathogen-Fungal Parasite-Undefined Saprotroph | 10 |
| Plant Pathogen-Plant Saprotroph | 7 |
| Fungal Parasite-Litter Saprotroph-Undefined Saprotroph | 5 |
| Animal Pathogen-Endophyte-Fungal Parasite-Lichen Parasite-Plant Pathogen-Wood Saprotroph | 3470177 |
| Animal Pathogen-Endophyte-Fungal Parasite-Plant Pathogen-Wood Saprotroph | 1939340 |
| Endomycorrhizal-Plant Pathogen-Undefined Saprotroph | 89270 |
| Animal Pathogen-Endophyte-Lichen Parasite-Plant Pathogen-Soil Saprotroph-Wood Saprotroph | 48554 |
| Bryophyte Parasite-Dung Saprotroph-Ectomycorrhizal-Fungal Parasite-Leaf Saprotroph-Plant Parasite-Undefined Saprotroph-Wood Saprotroph | 14770 |
| Animal Pathogen-Endophyte-Plant Pathogen-Wood Saprotroph | 13719 |
| Animal Pathogen-Endophyte-Plant Pathogen-Undefined Saprotroph | 2814 |
| Animal Pathogen-Dung Saprotroph-Endophyte-Lichen Parasite-Plant Pathogen-Undefined Saprotroph | 442 |
| Endophyte-Plant Pathogen-Wood Saprotroph | 266 |
| Animal Pathogen-Endophyte-Epiphyte-Plant Pathogen-Undefined Saprotroph | 210 |
| Animal Pathogen-Endophyte-Undefined Saprotroph | 93 |
| Endophyte-Lichen Parasite-Plant Pathogen-Undefined Saprotroph | 60 |
| Animal Pathogen-Endophyte-Epiphyte-Undefined Saprotroph | 52 |
| Dung Saprotroph-Endophyte-Plant Pathogen-Undefined Saprotroph | 40 |
| Endophyte-Plant Pathogen-Plant Saprotroph | 29 |
| Endophyte-Plant Pathogen-Undefined Saprotroph | 1 |
| Animal Endosymbiont-Animal Pathogen-Plant Pathogen-Undefined Saprotroph | 1 |
| Endophyte-Plant Pathogen | 4552095 |
| Endophyte-Epiphyte-Fungal Parasite-Insect Parasite | 122 |
| Lichen Parasite-Lichenized | 2 |
| Soil Saprotroph | 934687 |
| Undefined Saprotroph | 286361 |
| Leaf Saprotroph | 225876 |
| Plant Saprotroph-Wood Saprotroph | 126850 |
| Soil Saprotroph-Undefined Saprotroph | 6693 |
| Dung Saprotroph | 6206 |
| Dung Saprotroph-Soil Saprotroph-Wood Saprotrop | 1309 |
| Dung Saprotroph-Undefined Saprotroph | 232 |
| Dung Saprotroph-Plant Saprotroph | 131 |
| Wood Saprotroph | 122 |
| Dung Saprotroph-Soil Saprotroph | 12 |
| Ectomycorrhizal-Fungal Parasite-Plant Pathogen-Wood Saprotroph | 219231 |
| Endophyte-Litter Saprotroph-Soil Saprotroph-Undefined Saprotroph | 186205 |
| Dung Saprotroph-Ectomycorrhizal-Soil Saprotroph-Wood Saprotroph | 1044 |
| Endophyte-Undefined Saprotroph | 126 |
| Ectomycorrhizal-Undefined Saprotroph | 13 |
| Dung Saprotroph-Endophyte-Wood Saprotroph | 10 |
| Dung Saprotroph-Ectomycorrhizal-Litter Saprotroph-Undefined Saprotroph | 6 |
| Ectomycorrhizal | 528 |
| Endophyte | 268 |
| Orchid Mycorrhizal | 237 |
| Epiphyte | 18 |
| Arbuscular Mycorrhizal | 12 |
| Unassigned | 1719975 |

a
